# Key role of piRNAs in telomeric chromatin maintenance and telomere nuclear positioning in *Drosophila* germline

**DOI:** 10.1186/s13072-018-0210-4

**Published:** 2018-07-12

**Authors:** Elizaveta Radion, Valeriya Morgunova, Sergei Ryazansky, Natalia Akulenko, Sergey Lavrov, Yuri Abramov, Pavel A. Komarov, Sergey I. Glukhov, Ivan Olovnikov, Alla Kalmykova

**Affiliations:** 1 Institute of Molecular Genetics, Russian Academy of Sciences, Kurchatov sq. 2, Moscow, Russia 123182; 2 Department of Biochemistry, Faculty of Biology, Lomonosov Moscow State University, Moscow, Russia 119991

**Keywords:** Telomere, Germline, Retrotransposon, Transgene, piRNA cluster, HP1, Rhino, Chromatin, Transcription, *Drosophila*

## Abstract

**Background:**

Telomeric small RNAs related to PIWI-interacting RNAs (piRNAs) have been described in various eukaryotes; however, their role in germline-specific telomere function remains poorly understood. Using a *Drosophila* model, we performed an in-depth study of the biogenesis of telomeric piRNAs and their function in telomere homeostasis in the germline.

**Results:**

To fully characterize telomeric piRNA clusters, we integrated the data obtained from analysis of endogenous telomeric repeats, as well as transgenes inserted into different telomeric and subtelomeric regions. The small RNA-seq data from strains carrying telomeric transgenes demonstrated that all transgenes belong to a class of dual-strand piRNA clusters; however, their capacity to produce piRNAs varies significantly. Rhino, a paralog of heterochromatic protein 1 (HP1) expressed exclusively in the germline, is associated with all telomeric transgenes, but its enrichment correlates with the abundance of transgenic piRNAs. It is likely that this heterogeneity is determined by the sequence peculiarities of telomeric retrotransposons. In contrast to the heterochromatic non-telomeric germline piRNA clusters, piRNA loss leads to a dramatic decrease in HP1, Rhino, and trimethylated histone H3 lysine 9 in telomeric regions. Therefore, the presence of piRNAs is required for the maintenance of telomere chromatin in the germline. Moreover, piRNA loss causes telomere translocation from the nuclear periphery toward the nuclear interior but does not affect telomere end capping. Analysis of the telomere-associated sequences (TASs) chromatin revealed strong tissue specificity. In the germline, TASs are enriched with HP1 and Rhino, in contrast to somatic tissues, where they are repressed by Polycomb group proteins.

**Conclusions:**

piRNAs play an essential role in the assembly of telomeric chromatin, as well as in nuclear telomere positioning in the germline. Telomeric arrays and TASs belong to a unique type of Rhino-dependent piRNA clusters with transcripts that serve simultaneously as piRNA precursors and as their only targets. Telomeric chromatin is highly sensitive to piRNA loss, implying the existence of a novel developmental checkpoint that depends on telomere integrity in the germline.

**Electronic supplementary material:**

The online version of this article (10.1186/s13072-018-0210-4) contains supplementary material, which is available to authorized users.

## Background

Telomere transcription is an evolutionarily conserved feature of eukaryotic telomeres [1]. The biogenesis of telomeric transcripts has been shown to be tightly linked to telomere length control and formation of telomeric chromatin. Telomeric transcripts serve as precursors of small RNAs (tel-sRNAs) discovered in mammalian embryonic stem cells, in the ciliate *Tetrahymena thermophila*, in plants, and in *Diptera* [2–5]. Small RNAs generated by the subtelomeric regions in fission yeast, as well as certain tel-sRNAs, have been implicated in the assembly of telomeric heterochromatin [2, 5, 6]. Plant and mammalian tel-sRNAs are related to the class of Piwi-interacting RNAs (piRNAs) that are generated in the germline and in stem cells [7, 8]. However, the role of tel-sRNAs in germline-specific telomere function is poorly understood. Telomeric piRNAs and their role in telomere length control were first described in *Drosophila melanogaster* [3]. Using a *Drosophila* model, we performed an in-depth study of the biogenesis and function of the telomeric piRNAs in the germline.

The piRNA-mediated pathway provides silencing of transposable elements (TE) in the germline [7, 9]. In contrast to small interfering RNAs (siRNA), which are processed by the Dicer endonuclease from double-strand RNAs, piRNAs are generated from long single-stranded precursors. These piRNA precursors are encoded by distinct genomic regions that are enriched in damaged TE copies and termed piRNA clusters [10]. The dual-strand piRNA clusters found in the *Drosophila* germline produce piRNAs from precursors transcribed by both genomic strands. Distinct chromatin components of the piRNA clusters that couple transcription and RNA transport appear to direct the cluster-derived transcripts into the piRNA processing machinery [11–13]. The germline-specific homolog of HP1—Rhino (Rhi)—is essential for piRNA production from the dual-strand piRNA clusters [14–16]. piRNAs are required at early embryonic stages for deposition of Rhi and histone 3 lysine 9 trimethylation marks (H3K9me3) at the dual-strand piRNA clusters, but at later developmental stages, the chromatin of piRNA clusters is maintained by an unknown Piwi-independent mechanism [17].

Dual-strand piRNA clusters can be classified into several types according to their structure and location: extended pericentromeric TE-enriched regions [10], individual euchromatic TE copies [18], and transgenes which contain complementarity to endogenous piRNAs [19, 20]. In all cases, the clusters produce piRNAs that target complementary sequences located elsewhere in the genome.

The telomeres of *D. melanogaster* are maintained by transpositions of specialized telomeric retrotransposons, while the telomerase gene has likely been lost in an ancestor of *Diptera* [21]. The non-LTR *HeT*-*A, TART,* and *TAHRE* retroelements are organized in tandem head-to-tail telomeric arrays, with *HeT*-*A* being the most abundant [22–24]. Telomere-associated sequences (TASs) consist of complex satellite-like repeats and are located proximally to retrotransposon arrays. Analysis of the ovarian small RNA-seq data revealed abundant piRNAs corresponding to both genomic strands of telomeric retrotransposons and TAS [10]; thus, telomeric piRNA clusters can be formally related to the dual-strand type. However, the main feature of the telomeric piRNA clusters is that their transcripts serve both as a source of piRNAs and as their only targets simultaneously. Telomeric transcripts are processed into piRNAs that regulate telomeric TE expression and transposition rate onto chromosome ends in the germline [3, 25]. Violation of the balance between the levels of piRNAs and mRNAs encoded by telomeric retrotransposons can lead to disruption of telomere length control [3, 26]. It is clear that involvement in piRNA production should considerably affect telomere biology in the germline; however, telomeres have not been characterized as piRNA clusters to date. In previous studies, telomeric retrotransposons were generally included in the canonical set of TEs used to evaluate the impact of piRNA pathway mutation on TE expression and piRNA production. These data demonstrate that *HeT*-*A* and related *TAHRE* elements are extremely sensitive to piRNA pathway disruption showing up to 1000-fold overexpression in contrast to *TART*, which demonstrates only modest upregulation [3, 25, 27, 28]. Therefore, measurement of *HeT*-*A* expression has often been used as a readout of piRNA pathway disruption. However, *HeT*-*A* elements are not typical piRNA targets, since they are also the source of piRNAs. Several studies have indicated differences between transcriptional regulation of telomeric and non-telomeric piRNA clusters. Indeed, in contrast to *HeT*-*A*, the level of the piRNA precursors transcribed by non-telomeric piRNA clusters decreases upon piRNA pathway disruption [14, 15, 29].

Unique mapping of small RNA reads is the major source of information about the genomic origin of piRNAs; however, in the case of telomeres, it is technically challenging. Artificial sequences inserted into the endogenous piRNA clusters serve as unique marks that enable exploration of the highly repetitive genomic loci. Transgenic *Drosophila* strains carrying *P*-element constructs in the terminal retrotransposon array have been identified and characterized [30]. In contrast to the TASs that demonstrate Polycomb group (PcG) protein-mediated silencing of transgenes inserted in these regions [31–33], the telomeric retrotransposon arrays show euchromatic characteristics. Transgene reporters inserted in *HeT*-*A*–*TART*–*TAHRE* arrays are active in somatic tissues, which have allowed for selection of such transgenic strains [30]. Therefore, based on differing abilities to silence the integrated transgenes, two telomeric subdomains were defined within *Drosophila* telomere in somatic tissues, namely, transcriptionally active retrotransposon arrays and heterochromatic TAS [30, 31].

Taking advantage of the telomeric transgene insertions in combination with experiments on endogenous telomeric elements, we investigated piRNA production and chromatin structure of different telomeric loci in the ovaries of transgenic flies. It was shown that telomere-specific piRNAs significantly affect the chromatin structure and expression of different telomeric regions in the germline. In contrast to somatic tissues, the TAS and *HeT*-*A*–*TART*–*TAHRE* arrays show similar chromatin structure and transcriptional status in the germline and can be related to the piRNA-producing domain. At the same time, we found that piRNA production is not similar between the transgenes integrated in different telomeric retrotransposons. Chromatin and cytological studies provide convincing evidence that telomeric piRNA clusters are highly sensitive to piRNA loss in contrast to the heterochromatic non-telomeric dual-strand piRNA clusters. Moreover, piRNA loss causes telomere translocation from the nuclear periphery toward the nuclear interior. Our data, in combination with the previously observed differences in behavior of telomeric and non-telomeric piRNA clusters [17, 29, 34], suggest that a distinct type of piRNA cluster protects telomere integrity in the *Drosophila* germline.

## Results

### Transgenes located at different positions in telomeres produce small RNAs in *Drosophila* ovaries

**Fig. 1:**
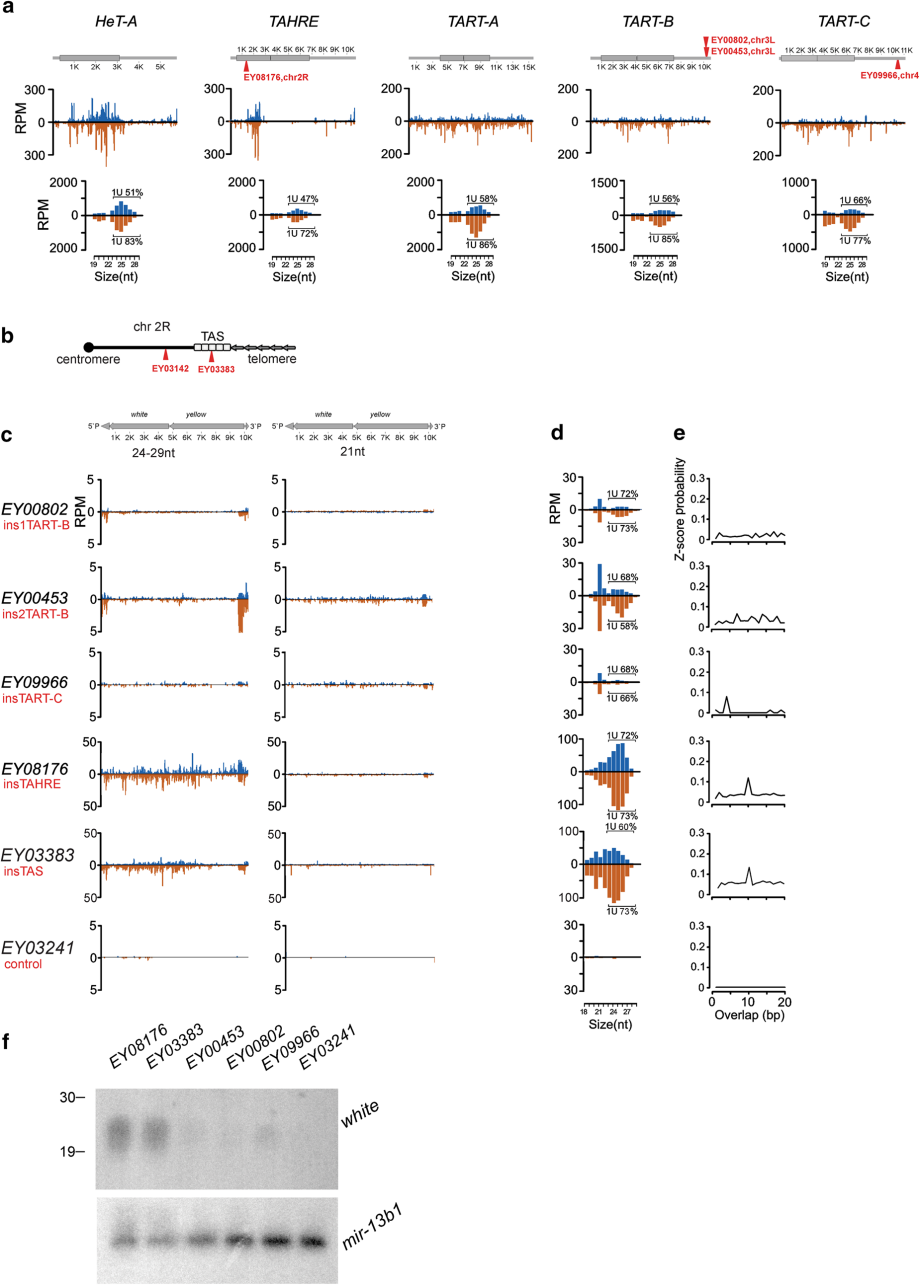
Generation of small RNAs by telomeric transgenes. **a** Schematic structure of telomeric elements is shown above. Insertion sites of transgenes are indicated as triangles situated above and below the schemes, which correspond to their genomic orientation. The profiles of small RNAs in ovaries of *yw* strain are shown along the canonical sequences of *HeT*-*A, TAHRE, TART*-*A, TART*-*B,* and *TART*-*C* telomeric retrotransposons. Normalized numbers of small RNAs (RPM, reads per million, 0–3 mismatches) in a 30-bp window were calculated. Length distribution of the telomeric element small RNAs is shown below. Percentages of reads having 1U are indicated for each strand (only 24–29-nt reads were considered). **b** Scheme of transgenic insertion sites in euchromatin and TAS of chromosome 2R. **c** Normalized numbers of small RNAs mapped to transgenic constructs (blue–sense; brown–antisense; no mismatches allowed). Mapping of piRNAs (24–29 nt) and siRNAs (21 nt) onto the transgenes is shown separately. Scheme of the P{EPgy2} transgene is shown above. Short names of telomeric insertions are indicated. **d** Length distribution of transgenic small RNAs. Percentage of reads having 1U are indicated for each strand (only 24–29-nt reads were considered). **e** Relative frequencies (Z-score) of 5′ overlap for sense and antisense 24–29-nt piRNAs (ping-pong signature). **f** Northern blot hybridization of the RNA isolated from the ovaries of EY08176, EY03383, EY00453, EY00802, EY09966, and EY03241 strains was done with the *white* riboprobe to detect antisense piRNAs. Lower panel represents hybridization to *mir*-*13b1* microRNA. P^32^-labeled RNA oligonucleotides were used as size markers

It is well known that transgenes inserted within TASs produce abundant piRNAs and exert piRNA-mediated silencing of the complementary targets [35–38]. However, the piRNA production capacity of transgenes located within telomeric retrotransposon arrays has not been investigated to date. In this study, we used four available transgenic EY strains on a *y*^*1*^*w*^*67c23*^ (*yw*) strain background carrying the P{EPgy2} construct in the telomeric retrotransposon arrays [30]. P{EPgy2} is a *P*-element-based vector containing *mini*-*white* and *yellow* genes. The transgene EY08176 was inserted into the GAG ORF of *HeT*-*A*-related *TAHRE* on chromosome 2R. The transgenes EY00453 and EY00802 were integrated into 3′ UTR of *TART*-*B1* on 3L, while the EY09966—into *TART*-*C* on the fourth chromosome. All *TART* insertions were located in the 3′UTR within the long non-terminal repeats containing the promoter region. Insertion sites are located between the sense and antisense transcription start sites [39], which seems to be a hot spot for insertions [30]. All transgenes were mapped at 12–23 kb from TASs [30]. The orange eye color of the EY08176, EY00453, and EY00802 transgenic flies corresponds to the previously reported phenotype and indicates a high level of the *mini*-*white* reporter gene expression [30, 33]. The EY03383 strain carries P{EPgy2} in 2R TAS [30]. The insertions in the TAS (EY03383) and in the telomere of the fourth chromosome (EY09966) are silenced and demonstrate a white or variegated eye color phenotype (Table 1). The euchromatic EY03241 transgene was used as a non-telomeric control. Insertion locations are shown schematically in Fig. 1a, b. DNA FISH on polytene chromosomes of salivary glands confirmed the telomeric localization of transgenes (Additional file 1: Figure S1). Short names of telomeric insertions are indicated in Fig. 1c.

**Table 1:** Comparison of telomeric transgene properties in somatic tissues and in the germline

Strain [30]	Insertion site	Somatic tissues [30, 31, 33]		Female germline					
		Eye color	Chromatin state	piRNA production	Rhi	HP1a	H3K9me3	Mini-white transcription	Chromatin state
EY08176	TAHRE Gag, chr.2R telomere	Orange	Active telomeric domain	+++	+++	+++	+++	+	Dual-strand piRNA cluster
EY00802	TART-B, 3′ regulatory region, chr.3R telomere			+	+	+++	+++	+	
EY00453				+	+	+++	+++	+	
EY09966	TART-C, 3′ regulatory region, chr.4 telomere	White	Repressed telomeric domains	+	+	+++	+++	+	
EY03383	TAS, chr.2R	Variegation		+++	++	+++	+++	+

Abundant endogenous *HeT*-*A, TAHRE,* and *TART*-specific small RNAs are found in *yw* and transgenic strains (Fig. 1a; Additional file 1: Figure S2); however, it is unclear how each particular telomeric element copy contributes to the production of piRNAs. To address this question, we sequenced small RNAs from ovaries of five telomeric transgenic strains and the EY03241 strain (control) with a euchromatic insertion. The euchromatic transgene EY03241 (control) produces a negligible amount of small RNAs (Fig. 1c, Additional file 2: Table S1). In contrast, all telomeric transgenes produce small RNAs; however, the levels vary considerably between transgenes (Fig. 1c), which may be attributed to variation in piRNA production between the integration sites. The small RNAs are mapped to both genomic strands of the entire transgene EY08176 (ins*TAHRE)*. Moreover, most of these small RNAs are 24–29 nt long and demonstrate 5′ terminal uridine bias (1U bias), which is a characteristic of the piRNAs (Fig. 1d). We found the sense/antisense piRNA pairs (relative to transgene) overlapping by 10 nt, which is a signature of the ping-pong piRNA amplification cycle [10, 40] (Fig. 1e). Such characteristics of transgenic small RNAs strongly suggest that the transgene is integrated within the pre-existing piRNA cluster. The EY03383 transgene inserted into the dual-strand piRNA cluster within the 2R TAS also produces abundant piRNAs from both genomic strands (Fig. 1c), similar to the transgenes integrated into subtelomeric piRNA clusters on the X and 3R chromosomes [19, 34, 38].

The EY00453, EY00802, and EY09966 (ins*TART*) transgenes produce fewer small RNAs compared to EY08176 (ins*TAHRE*) and EY03383 (insTAS) but apparently more than the EY03241 (control) transgene (Fig. 1c, Additional file 2: Table S1). A significant fraction of small RNAs produced by *TART* insertions are 21-nt siRNAs; no ping-pong signal was detected for the transgenic piRNAs showing 1U-bias. Interestingly, the production of 21-nt RNAs is less variable between the telomeric transgenes than that of piRNAs (Additional file 2: Table S1). Unique mapping of the small RNAs to all telomeric transgenes revealed single-mapped piRNAs derived from the P-element fragments and linkers, confirming that the observed effects are transgene-specific (Additional file 1: Figure S3).

Northern blotting of the *white*-specific small RNAs from the ovaries of transgenic strains confirmed the presence of abundant small RNAs in EY08176 (ins*TAHRE*) and EY03383 (insTAS) strains (Fig. 1f, Additional file 1: Figure S4).

Thus, all telomeric transgenes can be considered as piRNA clusters; however, the capacity to produce piRNAs varies significantly between the transgenes integrated into different telomeric sites, with *TART* promoter regions being considerably less productive (Table 1).

### HP1, Rhino, and H3K9me3 associate with different telomeric transgenes

**Fig. 2:**
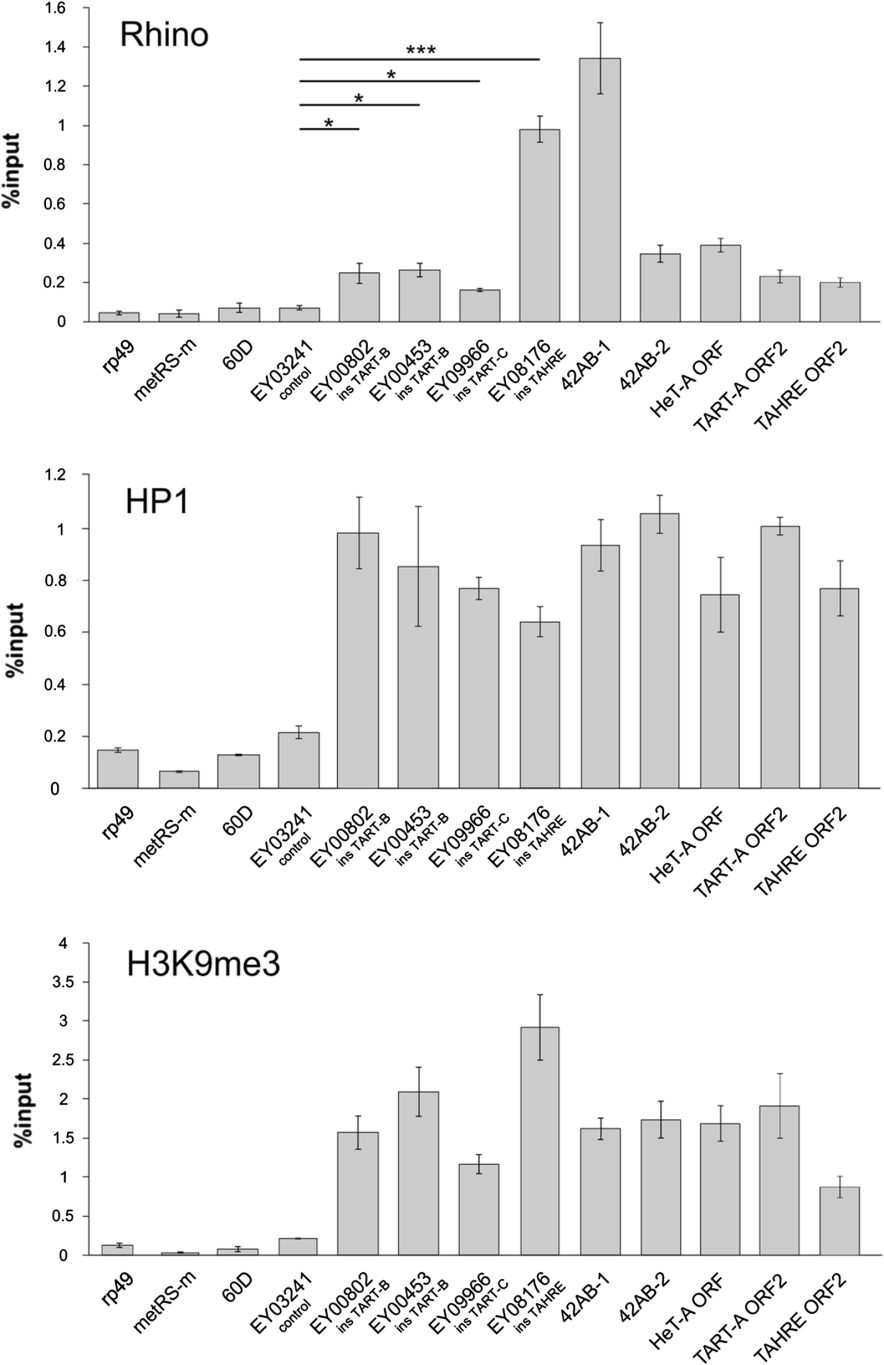
Chromatin components of the telomeric regions. HP1, H3K9me3, and Rhi occupancies at P{EPgy2} transgenes were estimated by ChIP-qPCR using primers corresponding to 5′-P-element transgenic sequence. Primers corresponding to ORFs were used for the analysis of endogenous *HeT*-*A, TART*-*A,* and *TAHRE.* Two regions of the *42AB* piRNA cluster are enriched in all studied chromatin components. *rp49, metRS*-*m,* and *60D* regions are used as negative controls. Asterisks indicate statistically significant differences in Rhi enrichment relative to EY03241 (control) (*P < 0.05 to 0.01, **P < 0.01 to 0.001, ***P < 0.001, unpaired *t* test). The difference in the HP1 binding between transgenes is statistically insignificant

The piRNA-guided transcriptional silencing is mediated by the deposition of HP1 and H3K9me3 [29, 41–43], whereas the germline-specific HP1 homolog Rhi serves as a chromatin marker of the dual-strand piRNA clusters [13–16, 44]. To answer the question if these chromatin components are associated with telomeric retrotransposons and transgenes in ovaries, we performed chromatin immunoprecipitation (ChIP) assay. The transcriptionally active *rp49* and *metRS*-*m* genes and the intergenic *60D* region were included in the analysis as negative controls. ChIP-qPCR using ORF-specific primers shows that endogenous *HeT*-*A, TAHRE,* and *TART* are enriched by HP1, Rhi, and H3K9me3 (Fig. 2). However, these data show the mean values for all telomeric copies and therefore do not reflect their possible heterogeneity. At the same time, transgene-specific primers enable us to examine the chromatin state at particular telomeric loci. Indeed, all studied transgenes were also considerably enriched in HP1 and H3K9me3, but we observed strong differences in Rhi binding among telomeric transgenes (Fig. 2). While Rhi occupancy was very high in two regions (5′ P-element arm and *mini*-*white*) of EY08176 (ins*TAHRE*), Rhi binding to *TART* transgenes was much less pronounced but statistically significant (Fig. 2; Additional file 1: Figure S5).

We conclude that telomeric retrotransposon arrays are heterogeneous in piRNA production, which correlates with Rhi binding. At the same time, all telomeric transgenes, regardless of the piRNA production rate and Rhi binding, associate with HP1 and H3K9me3 in ovaries. These observations raise the question regarding the role of the piRNA pathway in deposition of HP1 and H3K9me3, which are crucial for telomere function.

### piRNAs are required for deposition and maintenance of HP1, Rhi, and H3K9me3 chromatin components at telomeric retrotransposon arrays in ovaries

**Fig. 3:**
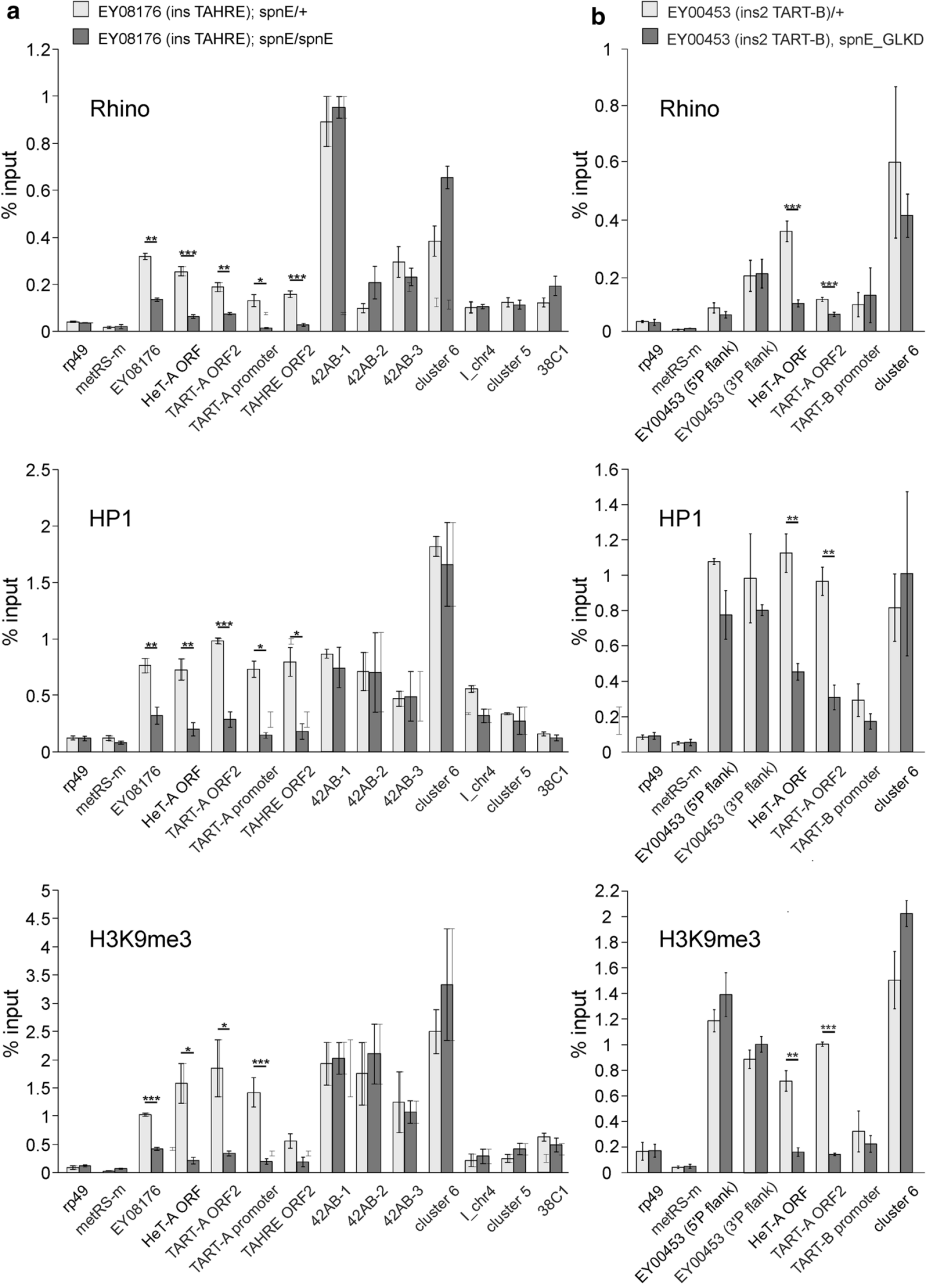
Role of piRNA pathway in the deposition of HP1, Rhi, and H3K9me3 at telomeric transgenes and endogenous telomeric retrotransposons in ovaries. **a** ChIP-qPCR analysis of HP1, Rhi, and H3K9me3 enrichment at EY08176 transgene (ins*TAHRE*), endogenous *HeT*-*A, TART*-*A, TAHRE* and a set of dual-strand piRNA clusters in ovaries of hetero- and trans-heterozygous (*spn*-*E*^*1*^*/spn*-*E*^*hls3987*^*) spindle*-*E* mutants. Asterisks indicate statistically significant differences in chromatin protein levels at the indicated regions between *spnE*/+ and *spnE/spnE* (*P < 0.05 to 0.01, **P < 0.01 to 0.001, ***P < 0.001, unpaired *t* test). **b** ChIP-qPCR analysis of HP1, Rhi, and H3K9me3 enrichment at EY00453 (ins2*TART*-*B*), endogenous *HeT*-*A, TART*-*B,* and dual-strand piRNA cluster 6 in ovaries upon *spnE* germline knockdown. Here, we used primers specific to the transgene insertion site instead of those to 5′P due to the presence of additional P-element-based constructs in the genome. The *TART*-*B* promoter was amplified using a primer pair surrounding the ins*TART-B* insertion

HP1 and H3K9me3 are important components of telomeric chromatin that are involved in the control of telomere length in different species including mammals [45]. HP1 and H3K9me3 are also present in the *Drosophila* telomeres in somatic tissues [31, 46, 47]; however, the mechanisms underlying their deposition at the telomere are not clear and likely differ between somatic and germline tissues. To study the role of the piRNA pathway in the deposition of HP1, H3K9me3, and Rhi at telomeres, we examined the association of these proteins with telomeric transgenes and endogenous telomeric repeats upon piRNA loss caused by depletion of the RNA helicase Spindle-E (SpnE) [3, 48]. In addition, we compared the chromatin dynamics of telomeric and non-telomeric piRNA clusters. We demonstrated that *spnE* mutation caused a considerable decrease in the association of HP1, Rhi, and H3K9me3 with the EY08176 telomeric transgene as well as with endogenous *HeT*-*A, TAHRE,* and *TART*-*A* elements accompanied by activation of their expression (Fig. 3a; Additional file 1: Figure S6). This result agrees with the previously observed loss of H3K9me3 and HP1 from *HeT*-*A* upon piRNA pathway disruption [28, 29]. In contrast to the telomeric regions, Rhi, HP1, and H3K9me3 are not displaced from *42AB* locus, *38C1,* and other dual-strand piRNA clusters in ovaries of *spnE* mutants (Fig. 3a). It is worth noting that different piRNA clusters use various mechanisms of transcriptional regulation. Transcription of most dual-strand piRNA clusters is initiated at random sites and regulated by non-canonical mechanisms. Several piRNA clusters, including *38C1*, use flanking promoters along with internal initiation sites for piRNA precursor transcription [15]. However, chromatin of the *38C1* cluster, as well as other non-telomeric clusters, is not changed in *spnE* mutant ovaries (Fig. 3a).

To examine the chromatin dynamics of the EY00453 transgene (ins2*TART*-*B*), we genetically combined it with the *spnE* short hairpin construct because the *spnE* locus is located on the same chromosome as the transgene insertion site, making it difficult to combine the transgene and *spnE* mutations. It is remarkable that the chromatin of this transgene is resistant to piRNA loss caused by *spnE* depletion (Fig. 3b), suggesting that the mechanism of chromatin maintenance at the regulatory region of *TART* is different from the other telomeric regions. We suggested that the *TART* promoter is resistant to piRNA-mediated chromatin assembly. We therefore performed ChIP-qPCR using primers specific to the endogenous *TART*-*B* promoter region in the vicinity of the ins*TART*-*B* transgene insertion site. These data show that chromatin in this region is also resistant to piRNA loss caused by *spnE* depletion (Fig. 3b). Surprisingly, the promoter regions of *TART*-*A* elements lose HP1, Rhi, and H3K9me3 in *spnE* mutants (Fig. 3a). The possible explanation for the differences between ChIP data on promoter and ORF regions of endogenous *TART*s related to different subfamilies is that *TART* copies are heterogeneous in chromatin structure and Rhi binding; however, the nature of this heterogeneity is still unclear and requires further investigation. Of note, the levels of Rhi vary at two different positions of *42AB*, which likely reflects the intrinsic heterogeneity of the chromatin structure of natural piRNA clusters.

Thus, ChIP data suggest that the piRNA pathway provides a germline-specific mechanism for HP1, Rhi, and H3K9me3 deposition at different telomeric regions. Additionally, it is essential for maintenance of this chromatin state during gametogenesis in contrast to the non-telomeric dual-strand piRNA clusters.

### piRNAs are required for telomere localization at the nuclear periphery but are dispensable for telomere capping and clustering in the germline

**Fig. 4:**
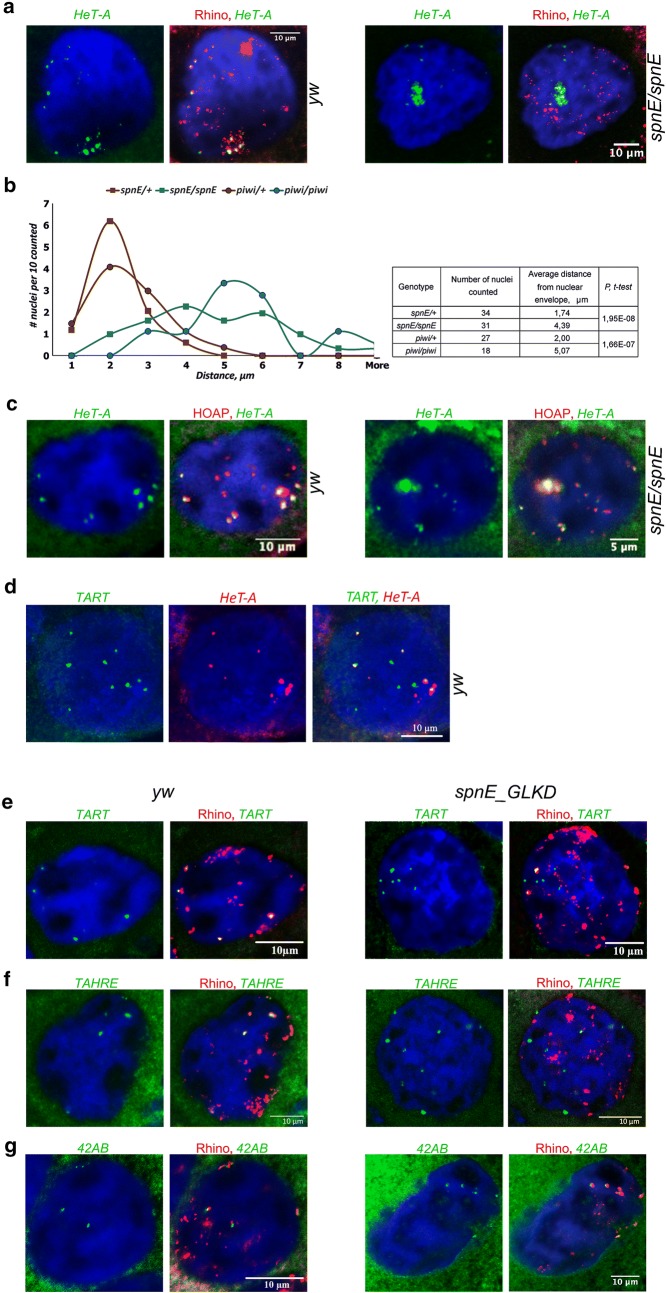
piRNAs are required for telomere localization at nuclear periphery. **a** DNA FISH with *HeT*-*A* (green) combined with Rhi staining (red) was performed on ovaries of the *yw* strain and of the *spn*-*E*^*1*^*/spn*-*E*^*hls3987*^ mutants. **b** Estimation of the positioning of clustered *HeT*-*A* signals relative to the nuclear surface of nurse cells by 3D quantitative confocal image analysis of *HeT*-*A* DNA FISH on ovaries of *spnE/*+, *spn*-*E*^*1*^*/spn*-*E*^*hls3987*^, *piwi*/+, and *piwi*^*2*^*/piwi*^*Nt*^ mutants. **c** piRNAs are dispensable for telomere capping and telomere clustering. DNA FISH with *HeT*-*A* probe combined with HOAP staining was performed on ovaries of the *yw* and *spn*-*E*^*1*^*/spn*-*E*^*hls3987*^ strains. **d** Double DNA FISH with *HeT*-*A* (red) and *TART* (green) probes was performed on ovaries of the *yw* strain. **e**, **f** piRNA pathway disruption causes loss of Rhi from *TART* but not from the *42AB* piRNA cluster. DNA FISH (green) with *TART-A* (**e**) or *42AB* (**f**) probes combined with Rhi staining (red) was performed on ovaries of the *yw* and *spn*-*E*^*1*^*/spn*-*E*^*hls3987*^ strains. DNA is stained with DAPI (blue). Nuclei of nurse cells from VIII to X stages of oogenesis are shown

To verify the association of Rhi with endogenous telomeres in wild type ovaries and upon piRNA loss, we visualized *HeT*-*A* and *TART* using DNA FISH combined with Rhi immunostaining. In contrast to the giant polytene chromosomes of salivary glands, the chromatids of highly polyploid nurse cells are only partially conjugated, allowing for detection of numerous DNA FISH signals. In the ovaries of the *yw* strain, most *HeT*-*A* foci are clustered and overlap with the largest Rhi foci, forming rosette-like structures near the nuclear envelope in the different *D. melanogaster* strains (Fig. 4a; Additional file 1: Figure S7a). We observed that the clustered *HeT*-*A* signals lose Rhi and are located toward the nuclear interior in *spnE* and *piwi* piRNA pathway gene mutants, although in *zucchini (zuc)* mutants these effects are less pronounced (Fig. 4a; Additional file 1: Figure S7b). According to previously published data, the same *zuc* mutation exerted a weak effect on the abundance of *HeT*-*A*-specific piRNAs compared to *piwi* and *spnE* [48], even though it is the most severe allelic combination of *zuc* [49]. Most likely, *zuc* function is less important in *HeT*-*A* chromatin assembly than that of *piwi* and *spnE*.

Positioning of the clustered *HeT*-*A* signals relative to the nuclear surface was estimated by 3D quantitative confocal image analysis of *HeT*-*A* DNA FISH samples on ovaries of the control, *spnE,* and *piwi* mutant flies. It was found that the distance from the center of the *HeT*-*A* FISH signal to the nuclear periphery of nurse cells increased significantly in *spnE* and *piwi* trans-heterozygous mutants compared to heterozygous controls (Fig. 4b).

Next, we addressed a question concerning the role of piRNAs in the deposition of the protective capping complex at the chromosome ends in the germline. We performed *HeT*-*A* DNA FISH combined with immunostaining of HOAP—the main component of the *Drosophila* telomere capping complex [50]—on ovaries of control flies and *spnE* mutants. HOAP extensively colocalizes with the clustered and individual *HeT*-*A* signals both in control and mutant nurse cell nuclei (Fig. 4c). Those HOAP signals that do not colocalize with *HeT*-*A* most likely correspond to telomeres lacking full-length *HeT*-*A* copies, since the *HeT*-*A* probe contains an ORF fragment. Previously, ChIP analysis showed a reduction of *HeT*-*A* enrichment by HOAP in *aubergine* and *armitage* but not in *ago3* and *rhi* piRNA pathway gene mutants [51]. Thus, HOAP loading at telomere ends appears to be mediated by the specific piRNA pathway components but not by piRNAs.

We suggested that the differences in chromatin structure and piRNA production between the telomeric transgenes might be explained by specific features of the telomeric retroelements in which they are inserted. Using dual color DNA FISH with *HeT*-*A* and *TART* probes corresponding to their ORFs, we showed that *HeT*-*A* and *TART* had different distributions in the nuclei of polyploid nurse cells. Surprisingly, in contrast to the clustered *HeT*-*A* foci, the majority of *TART* signals were separated, and only a few of them colocalized with *HeT*-*A* (Fig. 4d). Most likely, this pattern can be explained by the fact that the full-length *HeT*-*A* and *TART* are not present at all telomeres in the *yw* strain. In addition, *TART*-enriched telomeres do not seem to be involved in telomere clustering, in contrast to *HeT*-*A*-enriched telomeres. The *TART* DNA FISH combined with Rhi immunostaining demonstrates that the single *TART* signals colocalize with small individual Rhi foci (Fig. 4e). This pattern agrees with ChIP results showing that Rhi is deposited less at endogenous *TARTs* and *TART* transgenes than at *HeT*-*As*. *TAHRE* DNA FISH signals overlap with large Rhi foci (Fig. 4f). Depletion of *spnE* led to a dramatic decrease in the colocalization of Rhi with *HeT*-*A, TAHRE,* and *TART*-*A* DNA FISH signals in contrast to the *42AB* signals, and the latter continue to be associated with Rhi (Fig. 4; Additional file 2: Table S2).

Thus, piRNAs contribute significantly to the deposition of HP1, Rhi, and H3K9me3 at the telomeric *HeT*-*A*–*TART*–*TAHRE* arrays and to the nuclear position of telomeres in the germline. However, piRNAs play a minor role in the formation of the telomere capping complex and telomere clustering.

### Structure of subtelomeric chromatin in the germline

**Fig. 5:**
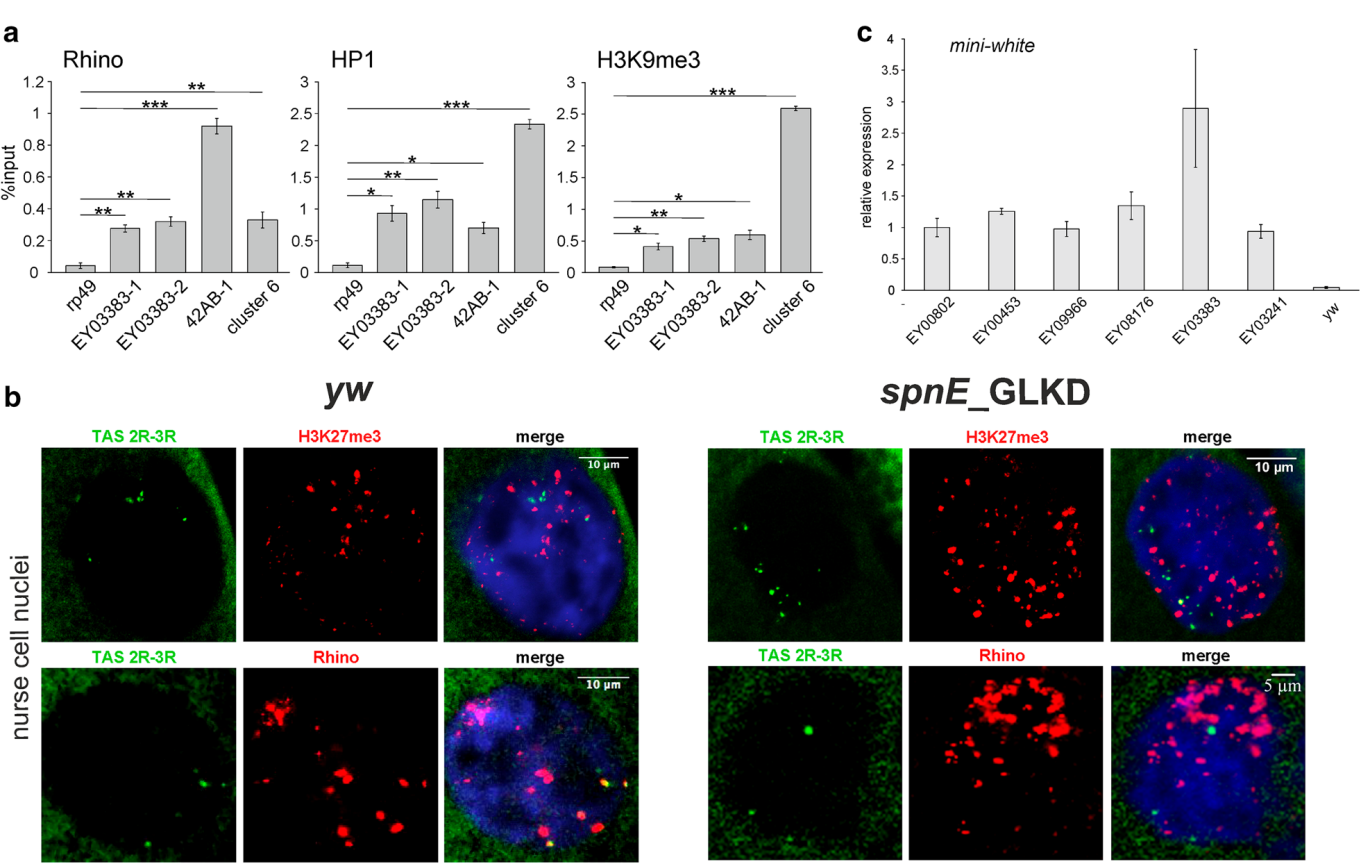
Comparison of subtelomeric chromatin structure in somatic and ovarian tissues. **a** HP1, H3K9me3, and Rhi occupancy at the EY03383 transgene located in the 2R TAS was estimated by ChIP-qPCR. *rp49* and *metRS*-*m* regions are used as negative controls. **b** DNA FISH with TAS 2R-3R probe (green) combined with Rhi (red) or H3K27me3 (red) staining was done on ovaries of *yw* and *spn*-*E*^*1*^*/spn*-*E*^*hls3987*^ strains. Nuclei of nurse cells (stage VIII–X) are shown. **c** RT-qPCR analysis of the expression levels of transgenic *mini*-*white* in ovaries of transgenic strains. *white*-specific primers detect only transgenic transcripts because endogenous *white* is partially deleted

*Drosophila* TAS regions consist of complex satellite-like tandem repeats. In the germline, these regions produce abundant piRNAs and are related to the most potent piRNA clusters [10]. On the contrary, in somatic cells, the *Drosophila* TASs are enriched in H3K27me3 marks and bind Polycomb group (PcG) proteins [31–33], which induces silencing of transgenic constructs inserted in TASs, a phenomenon known as the telomeric position effect [52, 53]. Thus, the chromatin structure of TAS regions is likely to be regulated in a tissue-specific manner. Using transgene-specific primers, we performed chromatin analysis of the EY03383 transgene (insTAS) in *Drosophila* ovaries and observed its enrichment in HP1, H3K9me3, and Rhi (Fig. 5a) which is in accordance with the piRNA production by this transgene (Fig. 1). To examine the chromatin state of endogenous TAS in the germline, we performed TAS DNA FISH combined with immunostaining of Rhi and H3K27me3—histone modification, associated with PcG silencing. The DNA probes corresponding to the 2R-3R and 2L-3L TASs were used for FISH on the ovaries of the control *yw* strain. TAS signals show a much stronger colocalization with Rhi than with H3K27me3 in the nuclei of nurse cells (Fig. 5b, Additional file 2: Tables S2, S3). We observed a loss of colocalization between Rhi foci and TAS signals in *spnE* mutants and upon *piwi* germline knockdown; at the same time, colocalization of H3K27me3 and TAS signals remained at a low level in the nurse cell nuclei of *spnE* mutants (Fig. 5b, Additional file 1: Figure S8). Thus, the PcG-dependent silencing of TAS is not established in the germline, which is in contrast to somatic tissues.

Next, we compared the expression activity of telomeric transgenes in ovaries. We found that the steady-state RNA levels of *mini*-*white* reporter gene were equally low in all transgenic strains but exceeded background signal in the *yw* strain in which *white* locus was partially deleted (Fig. 5c). Low levels of *mini*-*white* transcripts can be explained by a weak activity of the *white* promoter in the ovaries. Simultaneously, active expression of the *mini*-*white* reporter was observed in the eyes of EY08176 (ins*TAHRE*), EY00802 (ins1*TART*-*B*), and EY00453 (ins2*TART*-*B*) transgenic strains but not in EY03383 (insTAS) and EY09966 (ins*TART*-*C*) strains. Thus, the transcription activity of transgenes located at different positions within the telomere is similar in the germline but differs considerably in the somatic tissues and appears to be defined by the tissue-specific chromatin structure.

## Discussion

### piRNA production and Rhi binding differ along the telomeric region

In this study, we described telomeres as piRNA clusters using a combination of different approaches. The data on endogenous telomeric retrotransposons show that they produce piRNAs and associate with Rhi. Intriguingly, the piRNA production by individual telomeric transgenes depends on the type of telomeric retrotransposon in which the transgene is inserted. The transgene located within *TAHRE* produces considerably more piRNAs and shows stronger enrichment in Rhi than the transgenes located in the promoter region of *TART* elements. Accordingly, we revealed the low level of Rhi biding to the endogenous *TART*-*B* promoter region. Moreover, Rhi immunostaining and *TART* FISH data also demonstrate that much less Rhi is deposited on *TART* in comparison with *HeT*-*A*, suggesting a lower susceptibility of *TART* elements to engagement in piRNA production in general. Most likely, *TART* copies are not equivalent in their capacity for piRNA production and Rhi binding, although the nature of these differences is unclear. Previously published works have already underlined the strong differences between *HeT*-*A* and *TART* in genomic copy number, structure, patterns of transcription, and response to piRNA pathway disruption [3, 22, 54, 55]. *TART* transcripts are more stable [54], which can be explained by their role in providing reverse transcriptase (RT) for the transpositions of the main structural telomeric element *HeT*-*A* lacking RT. We suggested the intriguing possibility that the transcripts of full-length *TART* copies might be protected from piRNA biogenesis machinery by an unknown mechanism to ensure encoding of the crucial enzyme for telomere elongation, *TART* RT, in the germline.

Telomeric chromatin plays a pivotal role in telomere protection and maintenance. HP1 and H3K9me3 regulate capping, telomeric repeat silencing, and control of their transpositions onto chromosome ends [46, 47, 56]. Interestingly, all telomere insertions bind similar amounts of HP1 and H3K9me3 but strongly differ in Rhi association. Surprisingly, strong enrichment of the EY08176 transgene in Rhi, which recognizes the same H3K9me3 marks as HP1, does not abolish or significantly reduce HP1 binding compared to the *TART* insertions, indicating that Rhi and HP1 do not compete with each other for binding sites at telomeric chromatin. Thus, our data revealed heterogeneity in piRNA production and Rhi deposition within telomeric repeats. *TART* retrotransposons are less susceptible to Rhi binding than are *HeT*-*A* and *TAHRE*.

### Telomeric regions represent a distinct type of self-targeting dual-strand piRNA cluster

piRNA sources and piRNA targets are mainly represented by different genomic loci in the *Drosophila* germline. The piRNA clusters, enriched in damaged TE fragments, produce piRNAs that target active TEs [10, 29]. The telomeric piRNA clusters have a dual nature, possessing properties of both piRNA-clusters and piRNA-targets. It is well known that the piRNA targets are silenced at the transcriptional level via the assembly of repressive chromatin; loss of piRNAs causes strong reduction of HP1 and H3K9me3 marks at complementary targets, leading to their overexpression [25, 28, 29, 41, 42]. However, piRNA loss fails to activate non-telomeric piRNA cluster transcription and switching from a repressive to an active chromatin state [15, 17, 29].

In-depth analysis of the telomeric piRNA clusters revealed strong differences in chromatin dynamics between telomeric and non-telomeric piRNA clusters. Using different approaches, we demonstrated that the piRNA pathway mutations induce loss of HP1, H3K9me3, and Rhi from the telomeric transgene located in the *TAHRE*–*HeT*-*A* arrays, as well as from endogenous telomeric retrotransposons and TAS, in contrast to non-telomeric piRNA clusters. Of note, HP1 and H3K9me3 association with the EY00453 transgene (ins*TART*) is not affected by *spnE* depletion, indicating that the chromatin status of this insertion region or particular *TART* copies is maintained by piRNA-independent mechanism.

It has been shown that maternal and/or zygotic piRNAs were sufficient to induce formation of repressive chromatin at non-telomeric piRNA clusters in early embryogenesis and that this state was maintained during germ cell development, even upon piRNA loss at later developmental stages [17]. In contrast, piRNAs are required at all stages of germline development to maintain telomere silencing. Accordingly, it was also reported that piRNA production from the dual-strand *42AB* piRNA cluster was far less sensitive to germline depletion of Rhi or HP1a compared to the subtelomeric piRNA clusters and transgene located in this region [34]. Thus, the chromatin dynamics of telomeric retrotransposons are more similar to those of the piRNA targets than of the “canonical” dual-strand piRNA clusters. At the same time, the telomeric regions bind Rhi and produce piRNA precursors from both genomic strands and are thus attributed to the dual-strand piRNA clusters.

We propose that the fundamental difference between Rhi-dependent telomeric and non-telomeric piRNA clusters is due to their different transcriptional regulation. Strong bidirectional promoters drive transcription of the telomeric retroelements [39, 57, 58]. The loss of piRNAs causes activation of the promoters in telomeres resulting in switching from a repressive to an active chromatin state [25, 58]. In contrast, no discrete well-defined promoters were revealed within most of the heterochromatic non-telomeric piRNA clusters [59]. Transcription of certain clusters, such as *38C1*, is initiated not only from internal initiation sites but also from flanking promoters, which differ significantly from canonical promoters [15]. Accordingly, the chromatin of all piRNA clusters we tested, including *38C1,* is resistant to piRNA loss. The TATA box-binding protein (TBP)-related factor 2 (TRF2) and Moonshiner, a paralog of a transcription factor IIA (TFIIA), are required for non-canonical transcription initiation and piRNA production from most of the non-telomeric clusters [59]. In contrast, we described TRF2 as a strong repressor of *HeT*-*A/TAHRE* transcription, which is dispensable for *HeT*-*A* small RNA production [60]. In fission yeast, high levels of transcriptional activity at the siRNA target locus prevent heterochromatin assembly apparently through the displacement of the silencing complex [61], thereby indicating that the transcription status of the locus and nature of promoters are important factors that influence chromatin remodeling caused by small RNAs.

We found that the clustered *HeT*-*A* copies, normally positioned at the nuclear periphery, were located more toward the nuclear center following the loss of piRNAs. We suggest that this process is induced by the massive *HeT*-*A* overexpression and is related to the expression-dependent nuclear positioning phenomenon described by several groups (for review see [62]). The state of telomeres resulting from piRNA loss can be defined as telomere dysfunction. It is believed that various signaling mechanisms from dysfunctional telomeres can link telomere integrity and cell cycle regulation [63]. We suggest that telomere dysfunction caused by piRNA loss is directly linked to the developmental defects observed in piRNA pathway mutants.

Telomere clustering at the nuclear envelope is a commonly observed but not absolute phenomenon. Telomere clustering in close proximity to the nuclear periphery was observed in the *Drosophila* somatic cells [64, 65]. However, telomeres are not clustered but do associate with the nuclear envelope in *Drosophila* oocytes at the pachytene stage of meiosis [66]. We revealed clustering of the *HeT*-*A* DNA FISH signals next to the nuclear periphery in the nuclei of polyploid nurse cells; however, it is unclear which particular telomeres are involved in clustering. piRNA loss affects the peripheral localization of telomeres in the germline but does not alter telomere clustering or the assembly of the telomere capping complex.

**Fig. 6:**
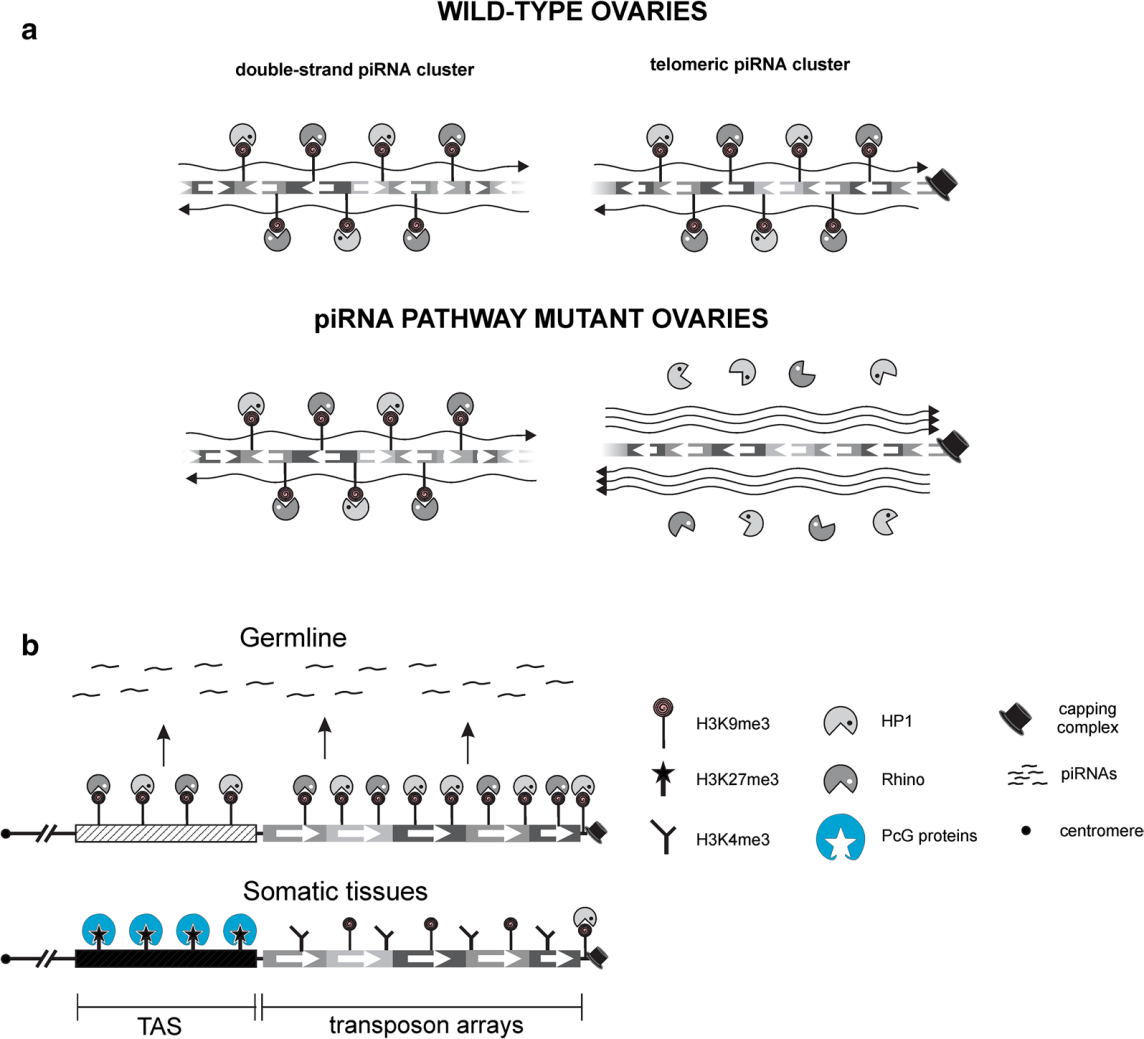
Telomeres represent a distinct type of self-targeting dual-strand piRNA cluster. **a** Schematic representation of three types of dual-strand piRNA clusters. The chromatin structure of “canonical” dual-strand piRNA clusters is established by maternally inherited piRNAs but maintained by a piRNA-independent mechanism. On the contrary, piRNAs are strongly required for maintenance of the chromatin state of telomeric and euchromatic TE-associated piRNA clusters during oogenesis. Assembly of telomere protection capping complex is not affected by piRNAs. **b** Comparison of telomeric chromatin in somatic and germ cells. A schematic distribution of chromatin components along telomeric retrotransposon arrays and TAS is based on our study and previously published results [30, 31, 33]. In somatic tissues, TAS and *HeT*-*A*–*TART*–*TAHRE* arrays are subdivided into repressed and transcriptionally active domains, respectively. In the germline, both telomeric regions form piRNA cluster(s) enriched in HP1, H3K9me3, and Rhi

In addition to the telomeric regions, some recently transposed transcriptionally active TE copies inserted into euchromatin produce piRNAs [18]. Strong reduction in the H3K9me3 and Rhi association upon *piwi* depletion is observed for such TE copies [15]. The main difference between the telomeric arrays and individual TE copies is that the latter are targeted by piRNAs, mainly produced by other piRNA clusters or TE copies. We conclude that the telomeric piRNA clusters constitute a specific type of Rhi-dependent actively transcribed piRNA clusters, which are highly sensitive to the presence of piRNAs (Fig. 6a).

### Germline-specific chromatin structure of *Drosophila* telomeres

Comparing the expression and chromatin structure of the telomeric transgenes in ovaries and somatic tissues reveals fundamental differences (Fig. 6b). Based on their ability to silence transgenes in somatic tissues, the TAS regions were defined as a heterochromatic domain, while the telomeric retrotransposon arrays were considered to be a transcriptionally active subdomain [30, 31]. Remarkably, the subtelomeric regions of diverse organisms consist of highly variable sequences that exert a silencing effect on transgenes integrated within these regions [67]. Thus, the conserved silencing capacity of TAS is presumably important for telomere function. We observed the similar chromatin properties of TAS and terminal *HeT*-*A*–*TART*–*TAHRE* arrays in the *Drosophila* germline. Both telomeric regions produce piRNAs, bind Rhi, and are expressed at a similar low level (Table 1). Our data raise an intriguing question about the competition, or developmentally regulated replacement, of different chromatin complexes at TASs. PcG protein binding sites were revealed in TAS repeats [32]. Indeed, immunostaining and genetic analysis of the PcG protein mutants clearly demonstrate that the TAS zone serves as a platform for PcG protein-mediated chromatin assembly in somatic tissues [31, 32]. We suggest that initiation of the piRNA precursor transcription in TAS displaces the PcG complexes or prevents their deposition in the germline. Our data agree with the previous observation that Piwi negatively regulates PcG protein binding to chromatin and H3K27me3 levels in *Drosophila* ovaries [68]. Tissue-specific silencing mechanisms have been observed by other groups; for example, the Polycomb repressive complexes were shown to silence transgenes carrying the retrotransposon *Idefix* in somatic tissues but not in ovarian follicular cells [69]. Interestingly, the retrotransposon *mdg1* copies marked by H3K27me3 in the ovarian somatic cells were not susceptible to the piRNA-mediated transcriptional silencing [42]. Our results and previously published observations indicate that complex and competitive relationships between the various chromatin complexes define the chromatin structure of the genomic loci, including telomeres, particularly in the developmental context.

## Conclusions

Our results demonstrate that the piRNA pathway is a robust mechanism of telomere homeostasis in the germline. piRNAs play a pivotal role in the establishment and maintenance of telomeric chromatin in the germline, facilitating loading of HP1 and H3K9me3 at different telomeric regions. piRNA pathway disruption results in telomere dysfunction characterized by the loss of heterochromatic markers, overexpression of telomeric repeats, and translocation of telomeres from the periphery to the nuclear interior. In contrast to somatic tissues, where TAS is a repressed domain, the *HeT*-*A*–*TART*–*TAHRE* arrays and TAS show similar chromatin structure and transcriptional status in the germline and belong to the Rhi-enriched piRNA-producing domain. However, strong heterogeneity in piRNA production and Rhi binding is observed along the telomeric transposon arrays. It is likely that *TART* retrotransposons are less susceptible to Rhi binding than *HeT*-*A.* The telomeric piRNA clusters belong to a specific type of Rhi-dependent piRNA clusters because telomeric retrotransposon transcripts driven by bidirectional promoters serve simultaneously as precursors of piRNAs and as their only targets. It is important that these transcripts are also used for telomere elongation in the germline.

## Methods

### *Drosophila* transgenic strains

Transgenic strains EY08176, EY00453, EY00802, EY09966, and EY03383 carrying the EPgy2 element and inserted within different telomeric regions were described previously [30] and were kindly provided by J. Mason. *Misy* natural strain was obtained from the collection of Institut de Genetique Humaine (CNRS), Montpellier, France. *P{EPgy2}Upf3*^*EY03241*^ (stock #16558) was obtained from the Bloomington Drosophila Stock Centre. Strains bearing *spindle*-*E* (*spn*-*E*) mutations were *ru*^*1*^ *st*^*1*^ *spn*-*E*^*1*^ *e*^*1*^ *ca*^*1*^/*TM3*, *Sb*^*1*^ *e*^*s*^ and *ru*^*1*^ *st*^*1*^ *spn*-*E*^*hls3987*^ *e*^*1*^ *ca*^*1*^/*TM3*, *Sb*^*1*^ *e*^*s*^. We used *piwi*^*2*^ and *piwi*^*Nt*^ alleles [70]. *Zuc* mutants were *zuc*^*Hm27*^*/Df(2L)PRL* trans-heterozygous flies [49]. GLKD (from “germline knockdown”) flies were F1 of the cross of two strains bearing constructs with short hairpin (sh) RNA (spnE_sh, #103913, VDRC; piwi_sh, #101658, VDRC) and strain #25751 (*P{UAS*-*Dcr*-*2.D}1, w*^*1118*^*, P{GAL4*-*nos.NGT}40*, Bloomington Stock Center) providing GAL4 expression under the control of the germline-specific promoter of the *nanos (nos)* gene.

Fluorescence in situ hybridization (FISH) with polytene chromosomes was performed as previously described [71]. A PCR fragment amplified using *white*-specific primers 5′-catgatcaagacatctaaaggc-3′ and 5′-gcaccgagcccgagttcaag-3′ was labeled with a DIG DNA labeling kit (Roche).

### RT-PCR analysis

RNA was isolated from the ovaries of 3-day-old females. cDNA was synthesized using random hexamers and SuperScript II reverse transcriptase (Life Technologies). cDNA samples were analyzed by real-time quantitative PCR using SYTO-13 dye on a Light Cycler 96 (Roche). Values were averaged and normalized to the expression level of the ribosomal protein gene *rp49*. Standard error of mean (SEM) for two independent RNA samples was calculated. The primers used are listed in Additional file 2: Table S4.

### Small RNA library preparation and analysis

Small RNAs 19-29-nt in size from total ovarian RNA extracts were cloned as previously described [38]. Libraries were barcoded according to Illumina TrueSeq Small RNA sample prep kit instructions and submitted for sequencing using the Illumina HiSeq-2000 sequencing system. After clipping the Illumina 3′-adapter sequence, small RNA reads that passed quality control and minimal length filter (> 18 nt) were mapped (allowing 0 mismatches) to the *Drosophila melanogaster* genome (Apr. 2006, BDGP assembly R5/dm3) or transgenes by bowtie [72]. Small RNA libraries were normalized to 1 Mio sequenced reads. The plotting of size distributions, read coverage, and nucleotide biases were performed as described previously [20]. Ovarian small RNA-seq data for *y*^*1*^*w*^*67c23*^ and transgenic strains EY08176, EY00453, EY00802, EY09966, EY03383, and EY03241 were deposited at the Gene Expression Omnibus (GEO), accession number GSE98981.

### Chromatin immunoprecipitation

For every IP experiment ~ 200 pairs of ovaries were dissected. ChIP was performed according to the published procedure [73]. Chromatin was immunoprecipitated with the following antibodies: anti-HP1a (C1A9, Developmental Studies Hybridoma Bank), anti-trimethyl-histone H3 Lys9 (Millipore), Rhi antiserum [58]. Primers used in the study are listed in Additional file 2: Table S4. Quantitative PCR was conducted with a Light cycler 96 (Roche). Standard error of mean (SEM) of triplicate PCR measurements for three-six biological replicates was calculated.

### FISH and immunostaining

The combined evaluation of protein and DNA localization was done according to the previously described procedure [71]. Rabbit anti-H3K27me3 (Abcam) and rat anti-Rhi antibodies [58] were used. The probes used for DNA FISH were: *TART*, cloned fragment of *TART*-*A* ORF2 corresponding to 434–2683 nucleotides in GenBank sequence DMU02279; *HeT*-*A*, cloned fragment of *HeT*-*A* ORF corresponding to 1746–4421 nucleotides in GenBank sequence DMU 06920; *TAHRE*, PCR fragment corresponding to 5147–6165 nucleotides in GenBank sequence AJ542581. The *TART* probe was labeled using a DIG DNA labeling kit (Roche), *HeT*-*A* by a Bio-Nick labeling system (Invitrogen), and *TAHRE* by PCR DIG labeling mix (Roche). Probes corresponding to 2R-3R TAS, 2L-3L TAS, and *42AB* regions were PCR fragments obtained with primers listed in Additional file 2: Table S4 and labeled with a PCR DIG DNA labeling mix (Roche). To stain DNA, ovaries were incubated in PBS containing 0.5 μg/ml DAPI. Three biological replicates were obtained for each experiment. Zeiss LSM 510 Meta and Olympus FV10i confocal microscopes were used for visualization. Confocal image z-stacks were generated with a slice step of 1.05 μM.

### Calculations of distance from the clustered *HeT*-*A* DNA FISH spots to the nuclear periphery

Calculations were performed using Imaris 7.4.2 software with manual segmentation of nuclei based on DAPI staining, automatic segmentation of in situ signal spots, and automatic calculation of a center of homogeneous mass corresponding to the main *HeT*-*A* cluster. FISH spot size is the diameter of a sphere encompassing all of the spots in the XY plane and Z position corresponding to the center of mass. The distance between the center of image masses and the nearest point on the nuclear surface was measured by increasing the radius of a sphere originated from the center of image masses until it intersected with the nuclear surface and later recording the radius as a distance. An independent two-sample *t* test was used to compare hetero- and trans-heterozygous mutants.

### Northern blot of small RNAs

Northern analysis of small RNAs was performed as previously described [20]. The *white* sense probe contained a cloned PCR fragment amplified using primers 5′-ctcacctatgcctggcacaatatg-3′ and 5′-attcagcagggtcgtctttccg-3′. Hybridization with P^32^ 5′-end-labeled oligonucleotide 5′-actcgtcaaaatggctgtgata-3′ complementary to the *miRNA*-*13b*-*1* was used as a loading control. The blots were visualized with a phosphorimager Typhoon FLA-9500 (Amersham). Northern blot quantification was done using ImageJ.

## Additional files

**Additional file 1: Figure S1.** Localization of telomeric transgenes. **Figure S2**. Profiles of telomeric retroelement small RNAs (related to Fig. 1a). **Figure S3**. Generation of small RNAs by telomeric transgenes (related to Fig. 1c). **Figure S4**. Quantification of Northern blots of small RNAs in transgenic strains (related to Fig. 1f). **Figure S5**. Rhi and HP1 occupancy at telomeric transgenes (related to Fig. 2). **Figure S6**. Expression of EY08176 telomeric transgene is increased in ovaries of the *spnE* mutants. **Figure S7**. Nuclear localization of telomeres. **Figure S8**. Subtelomeric chromatin in the germline (related to Fig. 5b).

**Additional file 2: Table S1.** Small RNA mapping to the telomeric and euchromatic transgenes. **Table S2**. Colocalization of *HeT-A*, *TART* and TAS with Rhino. **Table S3**. Colocalization of TAS with H3K27me3 in the *Drosophila germline*. **Table S4**. Primers used in the study (5’-to-3’).
