# Supplementary material for: Key role of piRNAs in telomeric chromatin maintenance and telomere nuclear positioning in Drosophila germline
Source: Epigenetics Chromatin. 2018 Jul 12;11:40. doi: 10.1186/s13072-018-0210-4 (PMC6043984; doi:10.1186/s13072-018-0210-4)
Supplement: Supplementary file 1 — Additional file 1: Figure S1. Localization of telomeric transgenes. Figure S2. Profiles of telomeric retroelement small RNAs (related to Fig. 1a). Figure S3. Generation of small RNAs by telomeric transgenes (related to Fig. 1c). Figure S4. Quantification of Northern blots of small RNAs in transgenic strains (related to Fig. 1f). Figure S5. Rhi and HP1 occupancy at telomeric transgenes (related to Fig. 2). Figure S6. Expression of EY08176 telomeric transgene is increased in ovaries of the spnE mutants. Figure S7. Nuclear localization of telomeres. Figure S8. Subtelomeric chromatin in the germline (related to Fig. 5b). [file 13072_2018_210_MOESM1_ESM.pdf]

Supplementary figures S1-S8

Key role of piRNAs in telomeric chromatin maintenance and telomere nuclear positioning in *Drosophila* germline

Elizaveta Radion, Valeriya Morgunova, Sergei Ryazansky, Natalia Akulenko, Sergey Lavrov, Yuri Abramov, Pavel Komarov, Sergey I. Glukhov, Ivan Olovnikov, and Alla Kalmykova

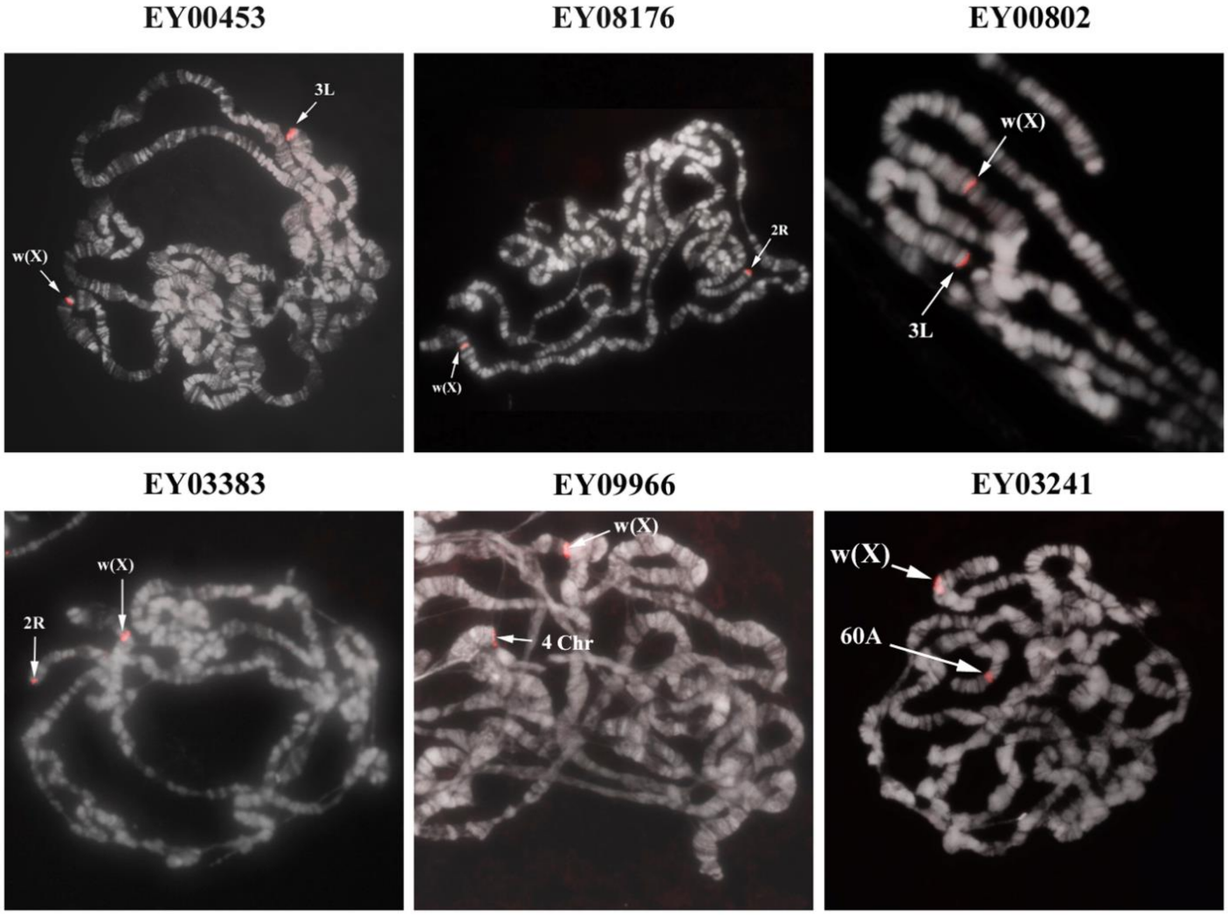

Figure S1. Localization of telomeric transgenes. DNA FISH with *white* probe on polytene chromosomes of salivary glands from EY08176 (insTAHRE), EY00453 (ins2TART-B), EY00802 (ins1TART-B), EY09966 (insTART-C) and EY03241 (control) *D. melanogaster* transgenic strains. Hybridization signals (red) are observed at telomeres and X chromosome corresponding to transgenes and endogenous *white* locus, respectively. EY03241 strain carries P{EPgy2} transgene in euchromatin. Chromosomes are stained with DAPI (grey).

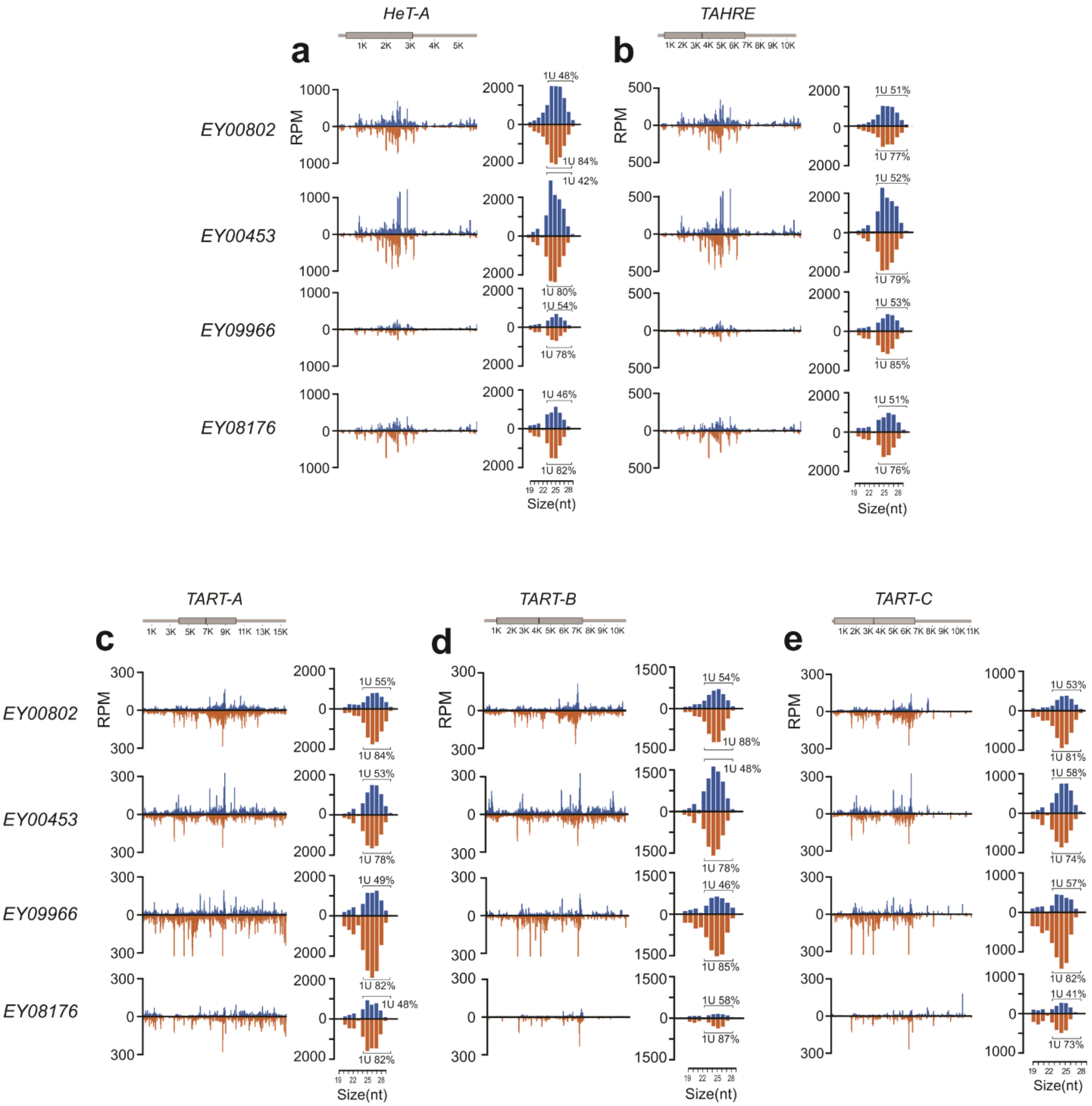

Figure S2. Profiles of telomeric retroelement small RNAs (related to Fig. 1a). The plots show normalized (in RPM) small RNAs from ovaries of EY08176 (insTAHRE), EY00453 (ins2TART-B), EY00802 (ins1TART-B) and EY09966 (insTART-C) mapped to the canonical sequences of *HeT-A* (A)*, TAHRE* (B) and *TART* (C-E) telomeric retrotransposons. The schemes of telomeric elements are shown above.

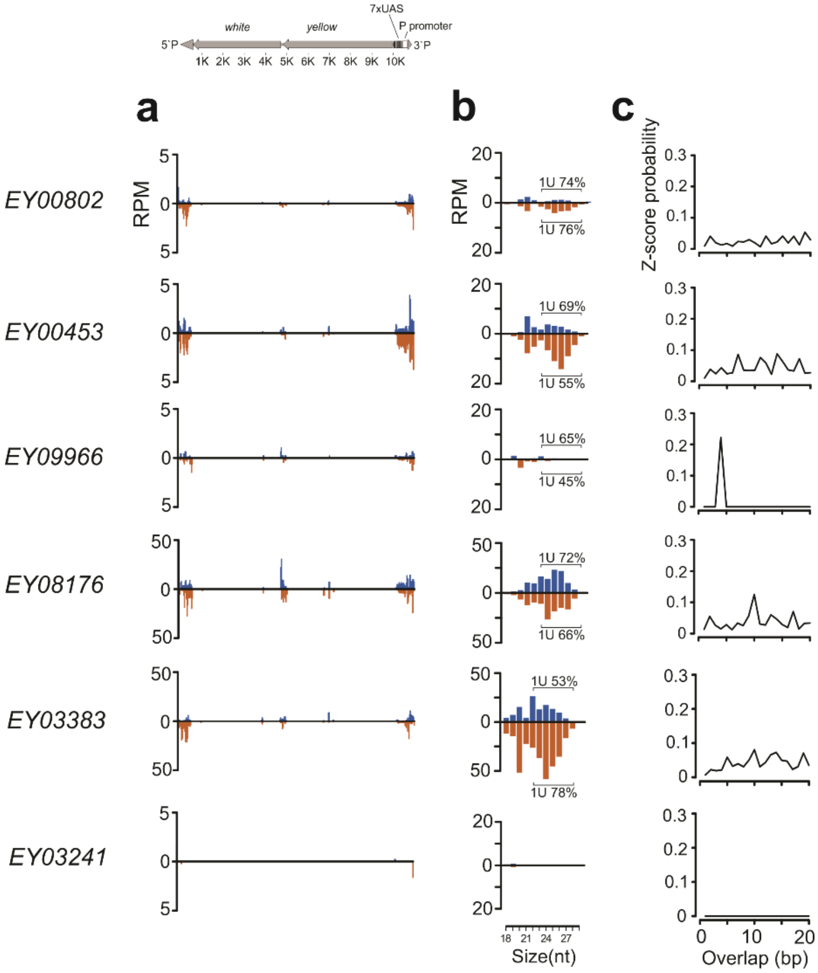

Figure S3. Generation of small RNAs by telomeric transgenes (related to Fig. 1c).

(A) Normalized numbers of single-mapped small RNAs mapped to EY08176 (insTAHRE), EY00453 (ins2TART-B), EY00802 (ins1TART-B) , EY09966 (insTART-C) and EY03241 (control) transgenic constructs (blue – sense; brown – antisense; no mismatches allowed). The scheme of P{EPgy2} transgene is shown above. Length distribution of small RNAs mapping to transgene (B) and ping-pong signal analysis (C) are shown.

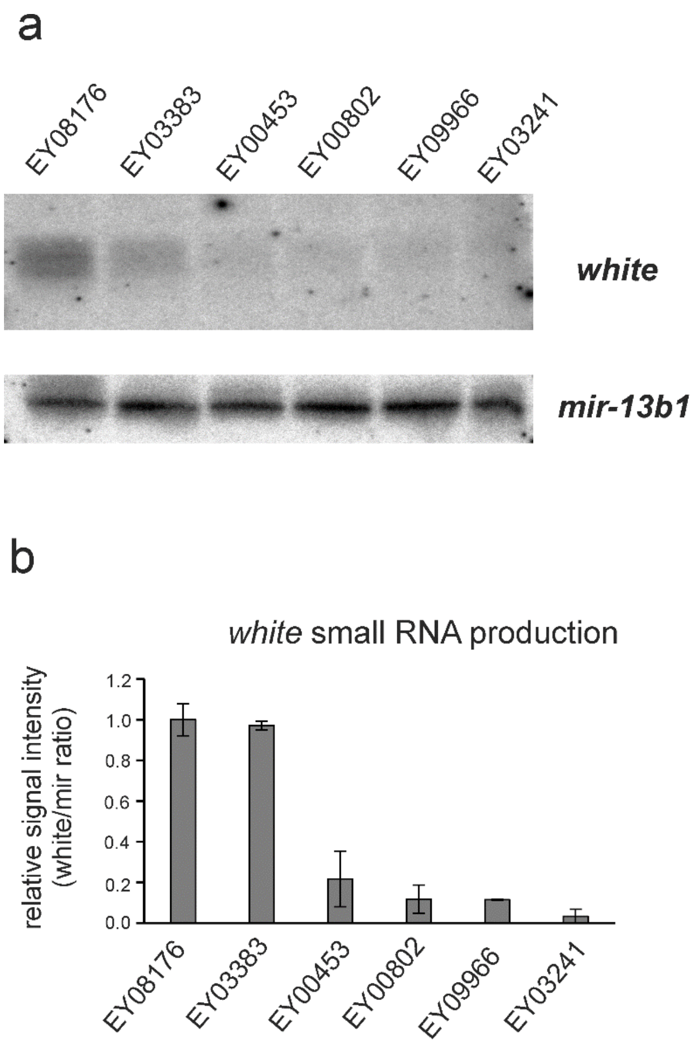

Figure S4. Quantification of Northern blots of small RNAs in transgenic strains (related to Fig. 1f). (A) Northern blot hybridization of small RNA isolated from the ovaries of EY08176 (insTAHRE), EY00453 (ins2TART-B), EY00802 (ins1TART-B), EY09966 (insTART-C) and EY03241 (control) strains was done with *white* riboprobe to detect antisense piRNAs. Lower panel represents hybridization to *mir-13b1* microRNA. (B) Quantification of Northern blots (Fig. 1f and S4a Fig.) showing the ratio of small RNAs in EY08176 versus other transgenic strains.

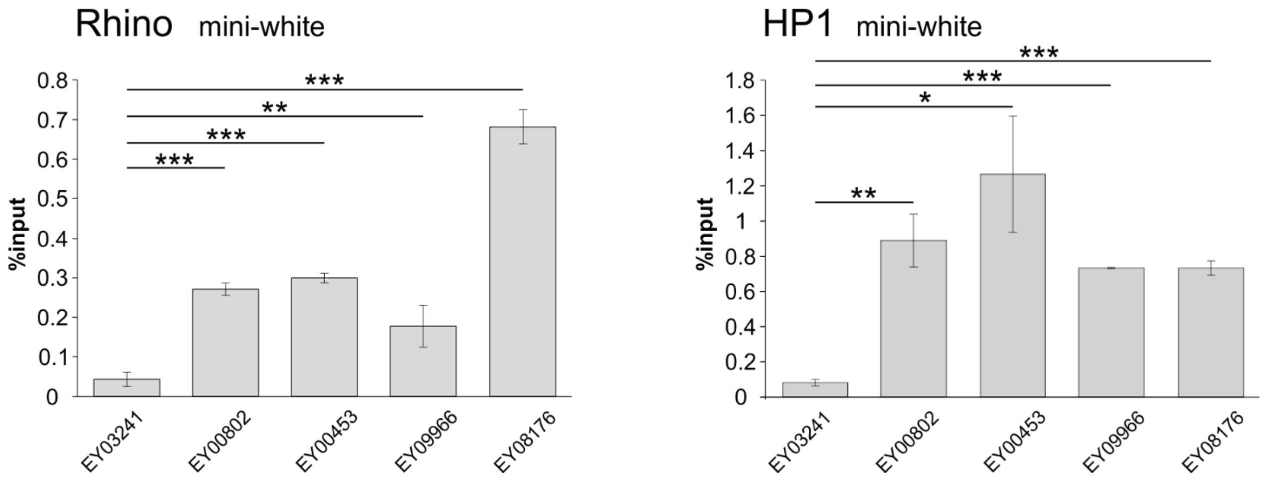

Figure S5. Rhi and HP1 occupancy at telomeric transgenes (related to Fig. 2). Rhi and HP1 ChIP-qPCR on ovaries of EY08176 (insTAHRE), EY00453 (ins2TART-B), EY00802 (ins1TART-B), EY09966 (insTART-C) and EY03241 (control) transgenic strains was performed using primers specific to *mini-white* transgenic sequence. Asterisks indicate statistically significant differences in Rhi or HP1 enrichment at the telomeric transgenes relative to EY03241 euchromatic transgene (* $P < 0.05$ to 0.01, ** $P < 0.01$ to 0.001, *** $P < 0.001$, unpaired t-test).

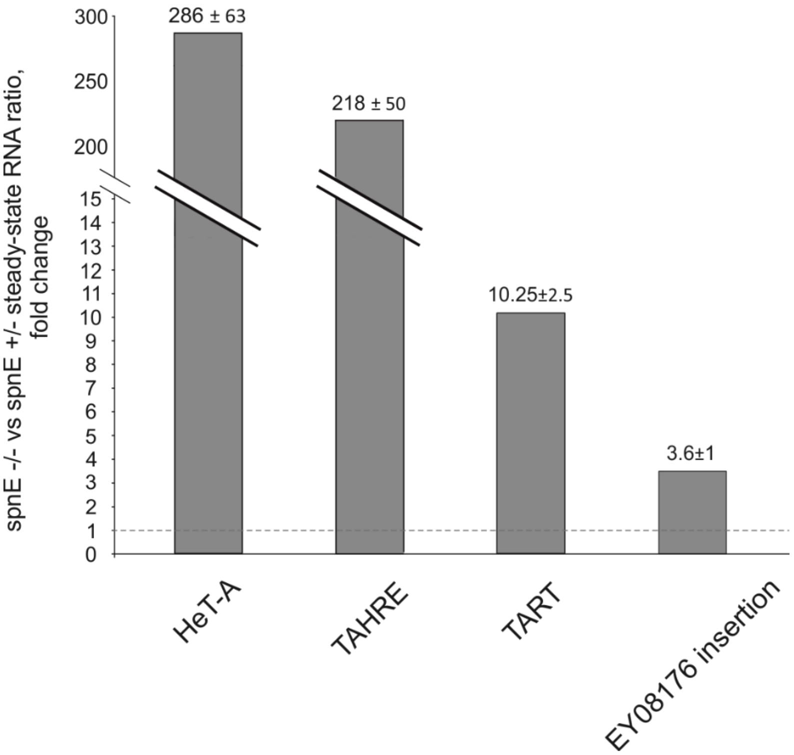

Figure S6. Expression of EY08176 (insTAHRE) telomeric transgene is increased in ovaries of *spnE* mutants. RT-qPCR analysis of the expression levels of endogenous *HeT-A, TART* and *TAHRE* retrotransposons and transgenic *mini-white* in ovaries of *spnE* mutants.

a

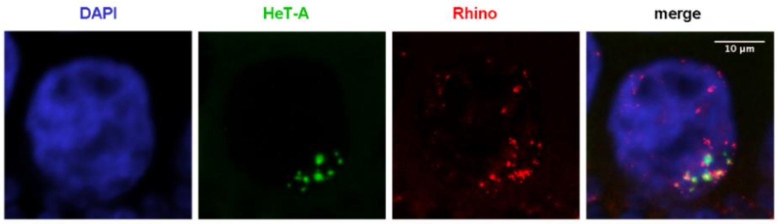

b

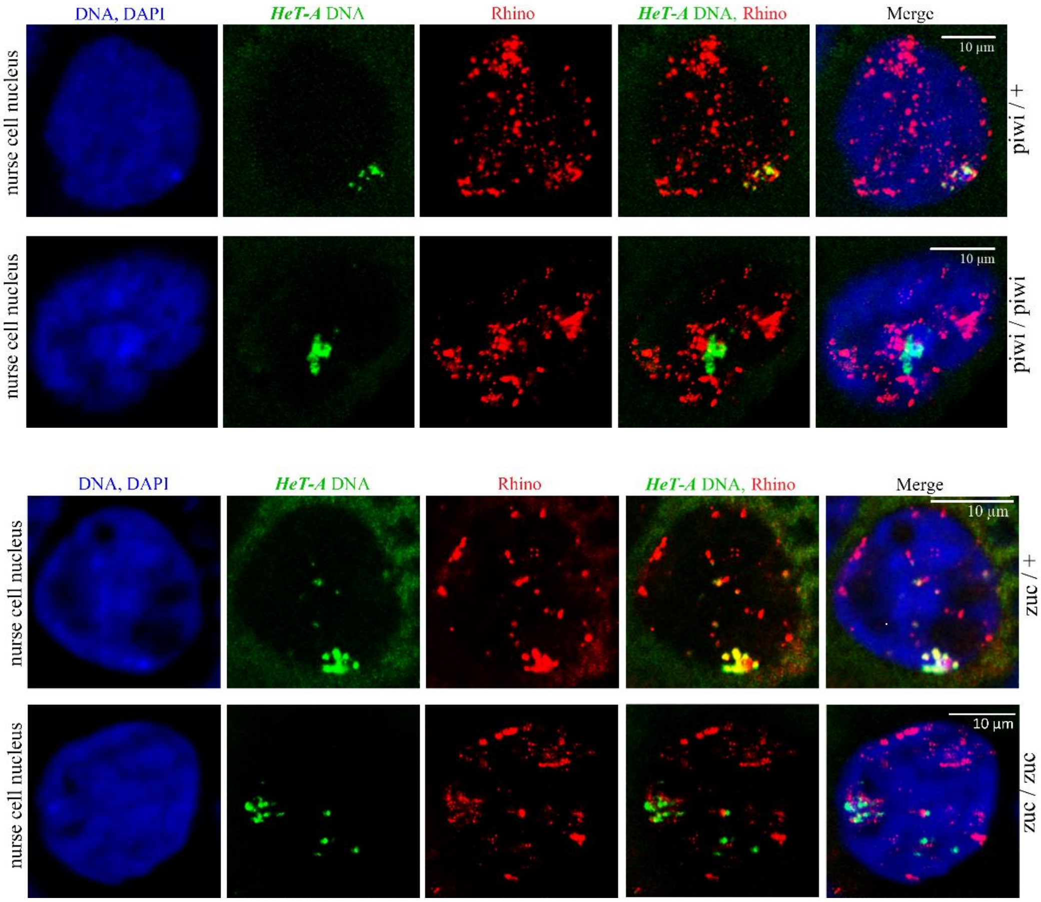

Figure S7. Nuclear localization of telomeres. (A) Endogenous *HeT-A* telomeric retrotransposons associate with largest Rhi domains. DNA FISH with *HeT-A* probe (green) combined with Rhi staining (red) was done on ovaries of *Misy* natural strain. Nurse cell nucleus of stage X of oogenesis is shown. (B) DNA FISH with *HeT-A* probe (green) combined with Rhi staining (red) was done on ovaries of $piwi^{2}/piwi^{Nt}$ and $zuc^{Hm27}/Df(2L)PRL$. *HeT-A* nuclear positioning and Rhi binding are considerably changed in *piwi* mutants, although in *zuc* mutants these effects are less pronounced.

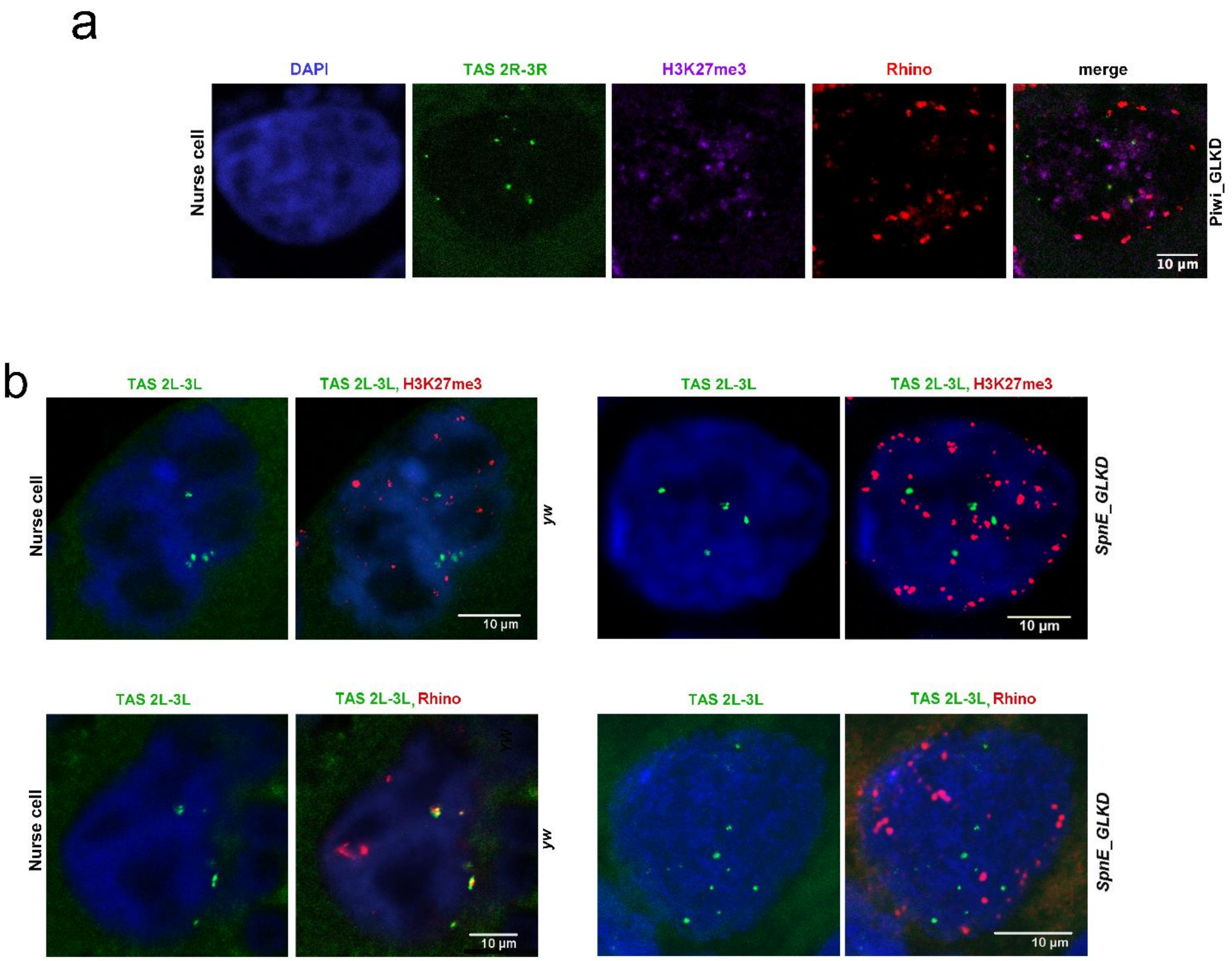

Figure S8. Subtelomeric chromatin in the germline (related to Fig. 5b). (A) DNA FISH with TAS 2R-3R probe (green) combined with Rhi (red) and H3K27me3 (magenta) staining was performed on ovaries upon *piwi* germline knockdown. Nurse cell nucleus at stage VIII is shown. (B) 2L-3L TAS signals do not associate with H3K27me3 mark in the germline**.** DNA FISH with TAS 2L-3L probe (green) combined with Rhi (red) or H3K27me3 (red) staining was done on ovaries of y^1 w^{67c23} strain. Nuclei of nurse cells (stages VIII-X) are shown.
