# Supplementary material for: Key role of piRNAs in telomeric chromatin maintenance and telomere nuclear positioning in Drosophila germline
Source: Epigenetics Chromatin. 2018 Jul 12;11:40. doi: 10.1186/s13072-018-0210-4 (PMC6043984; doi:10.1186/s13072-018-0210-4)
Supplement: Supplementary file 2 — Additional file 2: Table S1. Small RNA mapping to the telomeric and euchromatic transgenes. Table S2. Colocalization of HeT-A, TART and TAS with Rhino. Table S3. Colocalization of TAS with H3K27me3 in the Drosophila germline. Table S4. Primers used in the study (5’-to-3’). [file 13072_2018_210_MOESM2_ESM.pdf]

Table S1. Small RNAs mapping to the telomeric and euchromatic transgenes.

small RNA, reads per million (RMP)	strains					
	EY03241	EY09966	EY00453	EY00802	EY03383	EY08176
siRNA, 21 nt	1	18	71	20	199	211
piRNA, 24-29 nt	3	13	106	37	1114	2040
piRNA/siRNA ratio		0.7	1.5	1.9	5.6	9.7

Table S2. Colocalization of *HeT-A*, *TART* and TAS with Rhino.

Telomeric region	genotype	co-localization with Rhi, % of total number of signals	n, number of nurse cell nuclei (stages 8-10)
*HeT-A**	*yw*	96	35
	spnE -/-	14	33
TART-A	*yw*	93	42
	spnE -/-	4	32
TAS 2R-3R	*yw*	67	16
	spnE -/-	21	22
TAS 2L-3L	*yw*	86	11
	spnE -/-	18	43

*only individual non-clustered *HeT-A* signals were considered

Table S3 Colocalization of TAS with H3K27me3 in the *Drosophila* germline.

Telomeric region	Genotype, cell type	Co-localization with H3K27me3, % of total number of signals	n, number of nurse cell nuclei (stages 8-10)
TAS 2R-3R	*yw*	16	23
	spnE-/-	22	46
TAS 2L-3L	*yw*	2	20
	spnE -/-	9	17

Table S4. Primers used in the study (5'-to-3')

name/target	orientation	sequence
rp49	forward	ATGACCATCCGCCCAGC ATAC
	reverse	GCTTAGCATATCGATCCG ACTGG
metRS-m	forward	AAATCAACCGGCAAATT GTTTAGACG
	reverse	CACATAGTGCGAACTGTT GTAGCGAG
60D	forward	CCAGCCGAGACGAGCAC CATAAT
	reverse	TTCCCCATCCTCGAGCCC TG
5'P	forward	AGAGGAAAGGTTGTGTG CGGAC
	reverse	CTGCGAATCATTAAAGTG GGTATCA
EY08176 insertion	forward (TAHRE)	TGGCCTGGCTTGTGTTGG TG
	reverse (5'P)	TCCGCACACAACCTTTCC TCTCAAC
3'P	forward	TAATTCAAACCCCACGG ACA
	reverse	ATAACATAAGGTGGTCC CGTC
EY00453 insertion (5'P flank)	forward (TART)	GAGACCTGCTACCAAAA TGACCAG
	reverse (5'P)	TCCGCACACAACCTTTCC TCTCAAC
EY00453 insertion (3'P flank)	forward (3'P)	CGCTGTCTCACTCAGACT CAATACGAC AC
	reverse (TART)	GAT CCG GTC CGA TAG CCT AG
HeT-A ORF	forward	GGAGTGATGAGCGGCGG AAA
	reverse	CCAGGCAAGCGGACAAA CGA
TAHRE ORF2	forward	CATCAGACGAATCATAA ACGCC
	reverse	GATAAGGAGGTCATATA TTAAAGGG
TART-A ORF2	forward	AATGAACTTTGTCTGCCC TCCCA
	reverse	ATCTGTCTACTGTCCGCC TTCGCTA
TART-A promoter1	forward	GCTATTATCTTTTTTTTT GCCGCCA
	reverse	GATTTTTCTGCTTCGTTTC CGCTT
TART-B promoter	forward	GAGACCTGCTACCAA AATGACCAG
	reverse	GATCCGGTCCGATAG CCTAG

42AB-1	forward	CGTCCCAGCCTACCTAGTCA
	reverse	ACTTCCCGGTGAAGACTCCT
42AB-2	forward	TGTTTACCCAGAATGATGTTGAAATATAAGATG
	reverse	CACTGACTACGGTGCCTACAGCTATG
42AB-3	forward	CCGCATTGATTTGTAACGTGTAAC
	reverse	CCACACCCGGCCTCTAAAG
Cluster 6	forward	ACGGGACGACTGTTTGTGCTTGG
	reverse	GGCTTGTGGCTATGCTGGCGAA
I_chr4 (unannotated cluster)	forward	ACGGTCTCTCTGCTTTGCCTTG
	reverse	GACGGATTGCGGATTTATTTTGTT
Cluster 5	forward	ACAACACAGGGTTTGGCTTGTACC
	reverse	TGGAAAGCTTCCAAGAGTGCTCCA
38C1 left arm	forward	GATACTGGTTCTACGGTGCGAAAATAC
	reverse	GTGCTTGTGTGCTGTGTGAG
Mini-white	forward	CATGATCAAGACATCTAAAGGC
	reverse	AGTACCCCGAAGTATCCTAC
Mini-white 1st exon	forward	AGTTGCACTTTGTCAGCGGTTTCG
	reverse	CCCCTGCTTACCCACCCAAAA
42AB DNA FISH probe, Pld-1	forward	AGTCAAGTGCGTTTTGCTAGGCG
	reverse	GCCCGTTATCAAAATGTCCTCAGACCG
42AB DNA FISH probe, Pld-2	forward	CTGCCAGTTGAACTTGCCCATAAGC
	reverse	CCACCGACATCACCCTCAAAGC
2R-3R TAS probe	forward	CACACTGGCGTTCACTAC
	reverse	CATATTTCGCAACGTGACTG
2L-3L TAS probe	forward	CTCGTTCATCCGCCACCAT
	reverse	AGCACGACAGAGGAAGCAG
